# Medieval genomes from eastern Iberia illuminate the role of Morisco mass deportations in dismantling a long-standing genetic bridge with North Africa

**DOI:** 10.1186/s13059-025-03570-1

**Published:** 2025-04-28

**Authors:** Gonzalo Oteo-Garcia, Marina Silva, M. George B. Foody, Bobby Yau, Alessandro Fichera, Llorenç Alapont, Pierre Justeau, Simão Rodrigues, Rita Monteiro, Francesca Gandini, María Luisa Rovira Gomar, Albert Ribera i Lacomba, Josep Pascual Beneyto, Valeria Mattiangeli, Daniel G. Bradley, Ceiridwen J. Edwards, Maria Pala, Martin B. Richards

**Affiliations:** 1 School of Applied Sciences, University of Huddersfield, Queensgate, Huddersfield, HD1 3DH UK; 2 Dipartimento di Biologia Ambientale, Sapienza Università di Roma, Rome, Italy; 3 Centre for Palaeogenetics & Department of Archaeology and Classical Studies, Stockholm University, Stockholm, Sweden; 4 Ancient Genomics Laboratory, The Francis Crick Institute, London, UK; 5 Department of Prehistory, Archaeology and Ancient History, University of Valencia, Valencia, Spain; 6 Museu Arqueològic Municipal de la Vall d’Uixó, Vall d’Uixó, Spain; 7 Centro Arqueológico de l’Almoina & SIAM, Valencia, Spain; 8 Museu Arqueològic d’Ontinyent i la Vall d’Albaida, Ontinyent, Valencia, Spain; 9 Smurfit Institute of Genetics, University of Dublin, Trinity College, Dublin, Ireland

## Abstract

**Background:**

The Islamic influence on the Iberian Peninsula left an enduring cultural and linguistic legacy. However, the demographic impact is less well understood. This study aims to explore the dynamics of gene flow and population structure in eastern Iberia from the early to late medieval period through ancient DNA.

**Results:**

Our comprehensive genomic analysis uncovers gene flow from various Mediterranean regions into Iberia before the Islamic period, supporting a pre-existing pan-Mediterranean homogenization phenomenon during the Roman Empire. North African ancestry is present but sporadic in late antiquity genomes but becomes consolidated during the Islamic period. We uncover one of the earliest dated Islamic burials in Spain, which shows high levels of consanguinity. For the first time, we also demonstrate the persistence of North African ancestry in a Christian cemetery until the seventeenth century, in addition to evidence of slave trafficking from North Africa.

**Conclusions:**

This study reveals the complex interaction between political events and cultural shifts that influenced the population of eastern Iberia. It highlights the existence of a slave trade, underscores the low impact of the *Reconquista* in the genetic landscape, and shows the lasting impact of post-medieval events, such as the Expulsion of the Moriscos in 1609 CE, on the region’s genetic and cultural landscape, through mass population displacement and replacement.

**Supplementary Information:**

The online version contains supplementary material available at 10.1186/s13059-025-03570-1.

## Background

Andalusi Arabic was still spoken in the Iberian Peninsula in the seventeenth century CE, with its last remnants surviving in the hinterland of the Kingdom of Valencia [1]. This situation of social and linguistic isolation endured by part of the local population was the closing chapter of a story of acculturation that started nine centuries earlier. Although this process involved a degree of genetic admixture between newcomers and local Hispano-Romans [2], this admixture is not fully reflected in the present-day population [3].

Several ancient genomes have highlighted patterns of long-distance mobility in antiquity and medieval times across the Mediterranean [4–8]. Although some of these admixture events appear to have been transient [5, 6], their impact was almost as transformative as previous prehistoric migrations [9, 10]. Interestingly, however, their extent was more restricted in space, and most noticeable in urban centers [5–8]. This has become apparent as the field of ancient DNA (aDNA) started to shift the focus from continental scales to smaller scales, focussed on archeological questions in European contexts [11–18].

The genetic past of the Iberian Peninsula as a whole has been a subject of extensive research in the last few years, both through modern and aDNA [2, 3, 19, 20]. However, despite the significant amounts of data produced, the sampling effort has not been homogeneous across periods, due to research interests or sample availability [21], with the majority of studies focusing on prehistory [22–31]. The historical periods, although extensively studied by historiography, have only been observed cursorily through the lens of aDNA [2, 15, 18, 32].

The eastern lands of Spain from the river Ebro delta to Cape Nao, which today roughly comprise the majority of the Valencian territory, appear to have been a buffer zone between Greek colonies in the northeastern coast and Punic-Carthaginian colonies in the southeast [33]. Indigenous settlements did exist in the region, and some even gained temporary notoriety in the early phase of Roman Republican occupation. One such settlement was Arse, later known as Saguntum (modern Sagunto), whose siege by Hannibal Barca in 219 BCE triggered the start of the Second Punic War. Later developments include the foundation of the colony of *Valentia* (modern Valencia) in 138 BCE as one of the earliest outside Italy [34–37].

In the later Visigothic period, this land overlapped with the contested province of *Spania* under Byzantine occupation in the centuries prior to the Islamic conquest (552–624 CE). During this time, Valencia was a semi-autonomous city administered by bishops subordinate to the kings in Toledo [38, 39]. The territory was surrendered to the Islamic conquerors in the Treaty of Tudmir [40–42] by the local Visigothic governor [43, 44].

The Islamic conquest (711–756 CE) led to the establishment of Al-Andalus [45] and resulted in centuries of acculturation, co-existence, assimilation, linguistic intertwining, and conflict in the Iberian Peninsula.

Al-Andalus was initially shaped by a phase of Arab political dominion under the Umayyads during both Emirate (756–929 CE) and Caliphate (929–1031 CE) periods. Following the collapse of the Umayyad Caliphate, fragmentation ensued. The instability of the petty Islamic kingdoms during the Taifas periods led to distinct invasions by two North African empires between 1090 and 1237. As a result, Al-Andalus came to be dominated by successive Amazigh (Berber) military powers, the Almoravids (1040–1147 CE) and the Almohads (1121–1269 CE) [45].

The introduction of Islam resulted in a new societal organization among the local Visigothic and Hispano-Roman population [45]. In addition to a new Arab ruling minority, many Amazigh tribes came to settle in the Peninsula. This resulted in varied cultures and communities across the Iberian Peninsula: Christians living outside of Al-Andalus in the North, Christians living in Muslim territories of Al-Andalus (*Mozarabs*), and converts to Islam in Al-Andalus (*Muladis*), who came to represent the majority of the population.

By the end of the thirteenth century CE, the Christian kingdoms controlled the majority of Iberian territory. In the year 1238 CE, Valencia had been conquered and the new Kingdom of Valencia was founded by James I of Aragon. However, the *Muladis* still accounted for a large fraction of the population, and remained so instrumental to the economy in certain regions that the new Christian elites allowed them to maintain their cultural and religious traditions [46, 47], thus becoming the *Mudéjar*, i.e., Muslims living in Christian territories [48, 49]. However, by the sixteenth century CE, religious tensions against converts and *Mudéjars* had increased. As a result, in the first half of this century, the *Mudéjars* became targets of forced mass conversions, and the new converts to Christianity became known as *Moriscos* [50].

In practice, and often under draconian scrutiny, the *Moriscos* kept their language and customs, and many continued practicing their religion in secret [50, 51]. This was particularly true for *Moriscos* in the Kingdom of Valencia [1, 46, 52]. However, this dualism came to an end in 1609 CE, when Phillip III decreed the expulsion of all *Moriscos*. Estimates suggest that, in the Kingdom of Valencia, more than one third of the total population was expelled to North Africa [53]. The total numbers are estimated to have amounted to more than 300,000 people [54, 55].

Altogether, these historical episodes highlight the possibility for the arrival, admixture, and dwindling of varied sources of ancestry in Iberia in different historical periods [2]. Here we investigate in detail the extent to which North African-related ancestry fluctuated in eastern Iberia during historical times, with particular focus on the genetic impact of the establishment of Al-Andalus and later conquest of the region by Christian forces. To this end, we generated shotgun genome data from 12 individuals from various locations across the Valencian region, including three pre-Islamic burials (Roman and late Visigothic contexts), five Islamic inhumations, and four individuals from a Christian cemetery (both medieval and post-medieval), covering a period of over 1000 years.

## Results

### Time transect overview and uniparental diversity

**Fig. 1:**
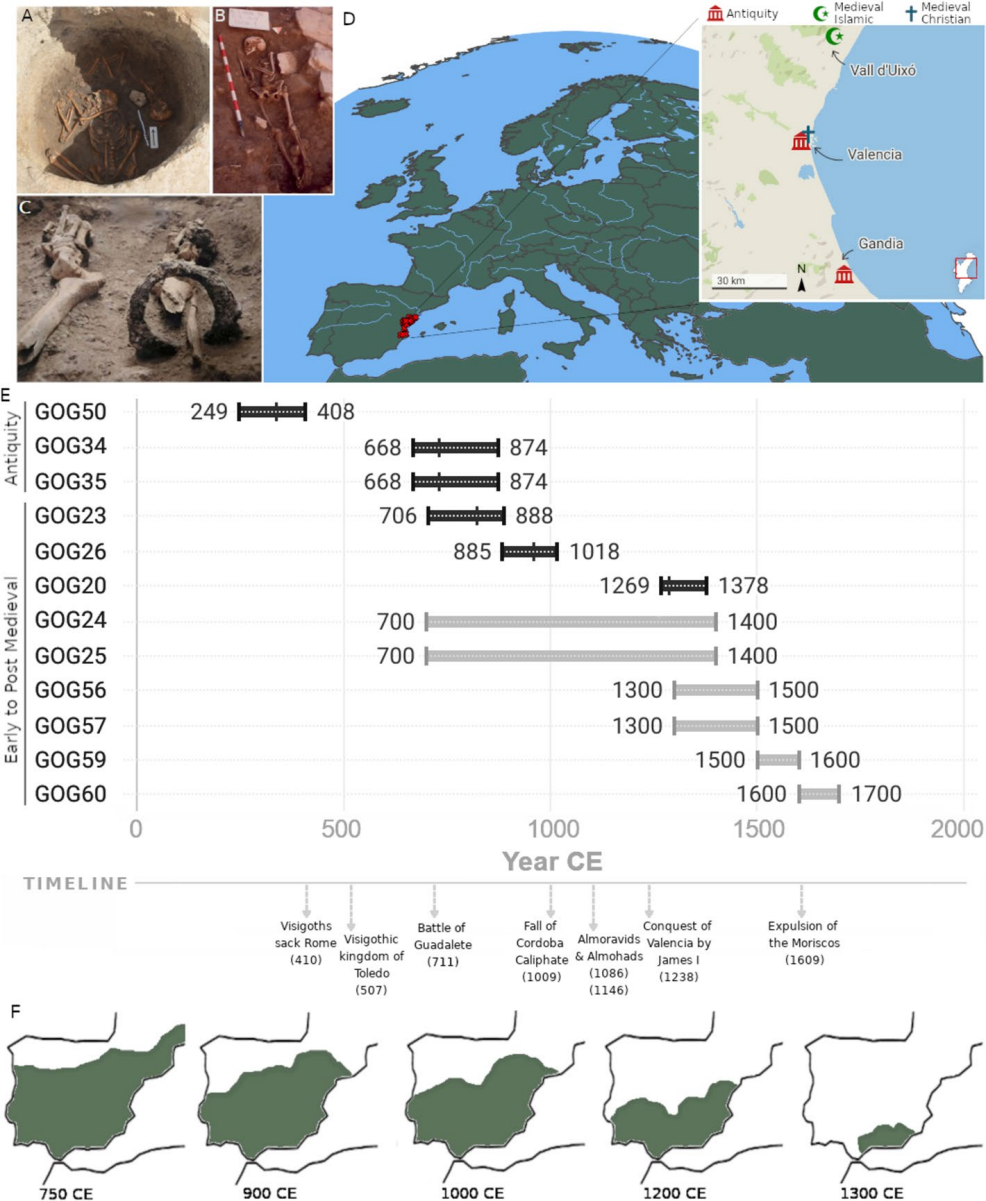
**A** Two kindred individuals from late antiquity contemporary to the Islamic arrival found in Gandía (GOG34 & GOG35). **B** Islamic burial (GOG25) from Vall d’Uixó. **C** Remains of individual GOG59 from Valencia, consisting solely of the lower extremities and an iron shackle on the right leg. **D** Map with the location of all samples screened (red crosses) in this Mediterranean region. Inset map details antiquity (Roman and post-Roman), Islamic and Christian (late and post-medieval) sites where the newly sequenced individuals in this study were found. **E** Ages for each individual studied above a timeline of relevant historical events. Black bars indicate calibrated radiocarbon dates, internal ticks indicate median calibrated age, and the range indicates 95% confidence intervals. Gray bars indicate relative dates based on stratigraphy, material culture, or excavation context. **F** Approximate extent of the borders of the territories of Al-Andalus (in green) from the eighth to the fourteenth centuries CE

We generated new historical genomes from 12 individuals (Table 1; Fig. 1A–C) from three sites in the Valencian region (Fig. 1D). The time transect (Fig. 1E) spans late antiquity (*n* = 3), medieval Islamic (*n* = 5), late medieval Christian (*n* = 2), and post-medieval Christian (*n* = 2) stages. This set of individuals contributes a novel shotgun-generated historical transect for Spain [56] (Additional file 2: Table S1).

**Table 1:** Overview of individuals sequenced in this study

Lab ID	Location	Period, culture	Radiocarbon dates	Genetic sex	mtDNA haplogroup	^a^ChrY haplogroup	^b^Depth of coverage
GOG50	Valencia, San Lorenzo	Antiquity, Roman	249–408 (cal.) CE	XX	D4e1	-	1.88x
GOG34	Gandía, Sanxo Llop	Antiquity, Post-Roman	668–874 (cal.) CE	XY	HV + 16311	R1b-L11	0.26x
GOG35	Gandía, Sanxo Llop	Antiquity, Post-Roman	668–874 CE^c^	XX	H2a1e1a	-	0.36x
GOG24	Vall d’Uixó, Benigafull	Medieval, Islamic	-	XY	U4a1d	J2a-M260	0.51x
GOG25	Vall d’Uixó, Ceneja	Medieval, Islamic	-	XX	L3d1	-	0.15x
GOG23	Vall d’Uixó, Benigafull	Medieval, Islamic	706–888 (cal.) CE	XY	HV	E1b-M183	0.63x
GOG26	Vall d’Uixó, Ceneja	Medieval, Islamic	885–1028 (cal.) CE	XX	H1	-	2.34x
GOG20	Vall d’Uixó, Benizahat	Medieval, Islamic	1269–1378 (cal.) CE	XX	J1c1b	-	0.47x
GOG56	Valencia, San Lorenzo	Late Medieval, Christian	-	XX	H + 7720	-	0.21x
GOG57	Valencia, San Lorenzo	Late Medieval, Christian	-	XX	R0a4	-	0.17x
GOG59	Valencia, San Lorenzo	Post-Medieval, Christian	-	XY	H5 + 152	E1b-M5081	0.26x
GOG60	Valencia, San Lorenzo	Post-Medieval, Christian	-	XY	K1a + 195	R1b-M269	0.23x

^a^Y-chromosome classification as per ISOGG 2019—most derived SNP

^b^Coverages reported in this table calculated after duplicate removal, mapping quality (MQ >20) and read length (bp >34) filters applied

^c^By association with GOG34 (same burial and first-degree relationship) but not directly dated

We selected these twelve individuals on the basis of DNA preservation from the total of 35 samples screened, which yielded a range of endogenous DNA from 0.01 to 41.58%, with the average endogenous DNA content being 7% (Additional file 1: Fig. S1; Additional file 2: Table S2) [57–60]. The sequencing of the DNA recovered from these 12 samples, which included petrous bones, molars, and a metatarsus, yielded average genome depth coverages between 0.21 × and 2.34 × (Table 1; Additional file 1: Fig. S1). Petrous bones performed better than teeth on average (Additional file 1: Fig. S1 and S2; Additional file 2: Table S2) [61].

We also report new radiocarbon dates (Additional file 2: Table S3) for three burials from antiquity and three medieval Islamic burials. To the best of our knowledge, the new dates uncovered one of the earliest Islamic burials (GOG23) in the Iberian Peninsula with genomic data (Fig. 1B), dating to 706–888 cal. CE (Additional file 2: Table S3). For the remainder of samples without radiocarbon dating, we used relative dating, based on archeological context, material culture, and other relevant information from excavation reports. The twelve individuals form the scaffold of a detailed genomic transect (Additional file 1: Fig. S1–S12; Additional file 2: Tables S4–S14) that starts in the third century CE and stretches to the seventeenth century CE (Fig. 1E).

The remains of the Roman burial (GOG50) from the city of Valencia had good endogenous DNA preservation (35%) in the context of this study. We identified the genetic sex [62] of this individual as female (Additional file 2: Table S15), carrying the mtDNA lineage D4e1. Notably, haplogroup D4e1 belongs to an East Asian clade that is rare in Europe throughout time [63, 64]. The radiocarbon dating of the sample indicates an age range of 249–408 cal. CE (median 338 CE), aligning well with its archeological context. These dates indicate the woman lived through a period of intense change in the Roman world marked by the Christian persecutions and the Edict of Milan (313 CE). However, although poorly preserved [56], the burial appears pagan in nature as it was accompanied by the remains of a swine head. Similar offerings are also found in much earlier burials in the city from the Republican period as part of the *porca praesentanea* ritual [65].

The remains of an adult (GOG34) and infant (GOG35) from the late Visigothic period in Spain (668–874 cal. CE) (Fig. 1A; Table 1) were found together in a round pit excavated in a site near Gandía (Fig. 1A, D). Genetic analysis identified the adult (GOG34) as male and the infant (GOG35) as female (Additional file 2: Table S15). They carried mtDNA haplogroups HV + 16,311 and H2a1e1a, respectively. The adult carried a R1b1a1b1a1a-L11 Y-chromosome (chrY) haplogroup. READ (Additional file 1: Fig. S3) detected a first-degree kinship between these two individuals. Combined with their ages at death, simultaneous burial, sex identification, and non-matching mtDNA haplogroups, we inferred the kinship status to be father–daughter. They were buried at the same time and although the cause of death is unknown, they bear no traumatic injuries [66].

From the medieval and later periods, we sampled various sites from Vall d’Uixo and the city of Valencia. Samples from Vall d’Uixó belonged to various Islamic cemeteries (*Maqbaras*) of rural settlements in the higher (Alquerías Ceneja and Benifagull) and lower (Alquería Benizahat) areas of the modern town [67]. As they expanded, these settlements eventually fused together [67]. We obtained older radiocarbon dates (706–888 and 885–1028 cal. CE, respectively) for individuals from the higher settlements (GOG23 and GOG26) (Fig. 1E), whereas the individual from the lower settlement was dated to a younger age (1269–1378 cal. CE). Some of these radiocarbon dates are much older than anticipated but confirmed a suspicion among local archeologists that the high ground settlement had been established earlier (Additional file 1: Fig. S11) [68, 69].

Among these samples, we found west Eurasian mtDNA haplogroups U4a1 d, HV*, H1, and J1c1b, as well as the more typically African haplogroup L3 d1. As for the chrY lineages, we found J2a1a1a2b2a1a-M92 and E1b1b1b1a1-M183 in the two males (Table 1). J2a1a1a2b2a1a-M92 likely has a Levantine origin [70, 71] but was present in Italy by Roman times [6]. E1b1b1b1a1-M183 is overwhelmingly the most common extant male lineage in North Africa, where it expanded within the last 3000 years [72].

The burials from a medieval Christian cemetery that remained in use until the mid-nineteenth century CE in Valencia were dated based on archeological context and historical record. Two of these (GOG56 and GOG57) were assigned as late medieval graves from the fourteenth to fifteenth centuries CE. We identified both these individuals as genetically female, with mtDNA haplogroups H + 7720 and R0a4. The others (GOG59 and GOG60) were post-medieval graves of two males dating to the sixteenth and seventeenth century CE, respectively. From the sixteenth century individual (GOG59), as seen in Fig. 1C, only the lower body was recovered with an iron shackle around the bones of his right leg still visible, suggesting enslavement. This individual carried a West Eurasian H5 mtDNA lineage and a North African E1b1b1b1a1-M183 chrY lineage. GOG60 carried a West Eurasian K1a mtDNA and a West Eurasian R1b1a1b-M269 chrY lineage (Table 1).

### Pan-Mediterranean genomic homogenization trends in the eastern coast of Roman Hispania

**Fig. 2:**
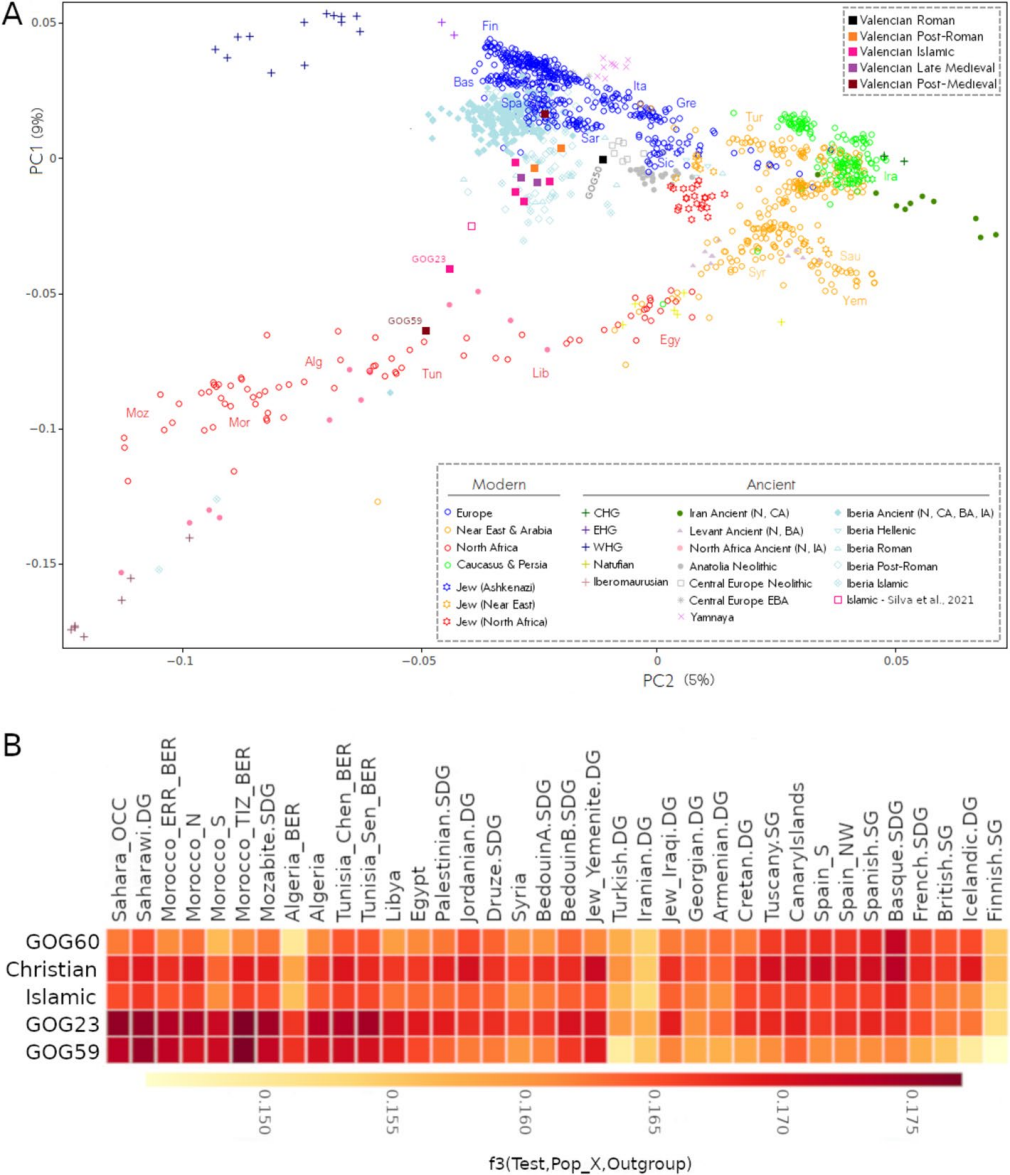
**A** PCA plot built with modern populations from around the Mediterranean world onto which key ancient and newly sequenced genomes from this study were projected. **B **Outgroup-*f3* test measuring shared drift of the groups defined by the ancient Valencian samples (Test) in this study with North African, Levantine, and European modern populations (Pop_X). The “Islamic” grouping includes GOG20, GOG24, GOG25, and GOG26. The “Christian” grouping encompasses GOG56 and GOG57

All three samples from antiquity appear as significant outliers with respect to the modern Spanish population in the PCA (Fig. 2A).

The Roman female individual (GOG50) trends toward modern central and eastern Mediterranean populations, sitting midway between present-day Spanish and Sicilian individuals (Fig. 2A). Her heterozygosity levels appear slightly higher than the average, however (Additional file 1: Fig. S8), suggesting multiple ancestry contributions from within the Mediterranean world (Additional file 1: Fig. S7 and S8, S10 A; Additional file 2: Table S12). In the PCA, this individual shares a hybrid space defined by some other Roman Iberian and Punic individuals from Sardinia [8]. We conclude that her affinities were derived from a complex admixture process of two genetic sources that do not exist today as consolidated populations. One parental source was probably Iberian, but with higher North African ancestry than the present-day Spanish average [2, 3]. The other source was likely of Italian-Sardinian origin, with possible eastern Mediterranean influences, typical in the Roman Imperial period [6]. However, it is difficult to pinpoint the exact sources, due to the extremely high heterogeneity observed among individuals in the Mediterranean during late Roman times [6–8]. Proximal *qpAdm* modelling also highlights the contribution of an eastern Mediterranean source, with 62.4–70.3% of her ancestry modelled as related to *Lebanon_Roman.SG*, albeit with large SEs (*p*-values: 0.08 and 0.06 and SEs ranging from 38.3 to 13.4%) (Additional file 2: Table S13). This is consistent with an increased *Balkan_N* admixture proportion observed in the distal *qpAdm* model (68.6 ± 3.8%, *p*-value = 0.238) (Additional file 2: Table S14).

Furthermore, the mitochondrial haplogroup D4e1 carried by GOG50 is extremely rare among ancient Europeans and Near Easterners [73]. There are only a couple of instances in present-day Europeans. Modern data show that D4 and D4e lineages (except D4e1) are all found in East Asia. Other D4e1 sister haplotypes are also widely dispersed in Central, East, and Southeast Asia and, to a minor degree, in South Asia [64].

In particular, haplogroup D4e1 has been identified in a Xiongnu individual (TAK008) from the Altai, dating to 51–155 BCE [74], while it is not found among 60 Xiongnu individuals from further east [75]. The Xiongnu formed a diverse steppe empire, which might be a potential source for the lineage in Europe through third century CE dispersals of Huns, Alans, and Sarmatians [76]. It has also been identified in a burial of similar age (203–319 cal. CE) from the Roman era in Turkey [71]. Our discovery of the lineage in Iberia, so far west in the Mediterranean, appears strikingly early. However, as shown in García Borja et al. [11], migrants from Eastern Europe were already passing through Hispania before the start of the so-called Germanic invasions.

To trace recent East Asian ancestry in the genome of this individual, we made use of *f*-statistics and local ancestry inference (LAI). Outgroup-*f3 and f4* statistics did not detect extra genetic affinity to Asian populations (Additional file 2: Table S12). Similarly, LAI evaluation with RFmix did not reveal outstanding stretches of East Asian-related ancestry, although it shows some very short South Asian-like haplotypes (Additional file 1: Fig. S9). This result mirrors the finding in the Xiongnu D4e1 individual (TAK008) who already carried around 90% West Eurasian ancestry [74].

**Fig. 3:**
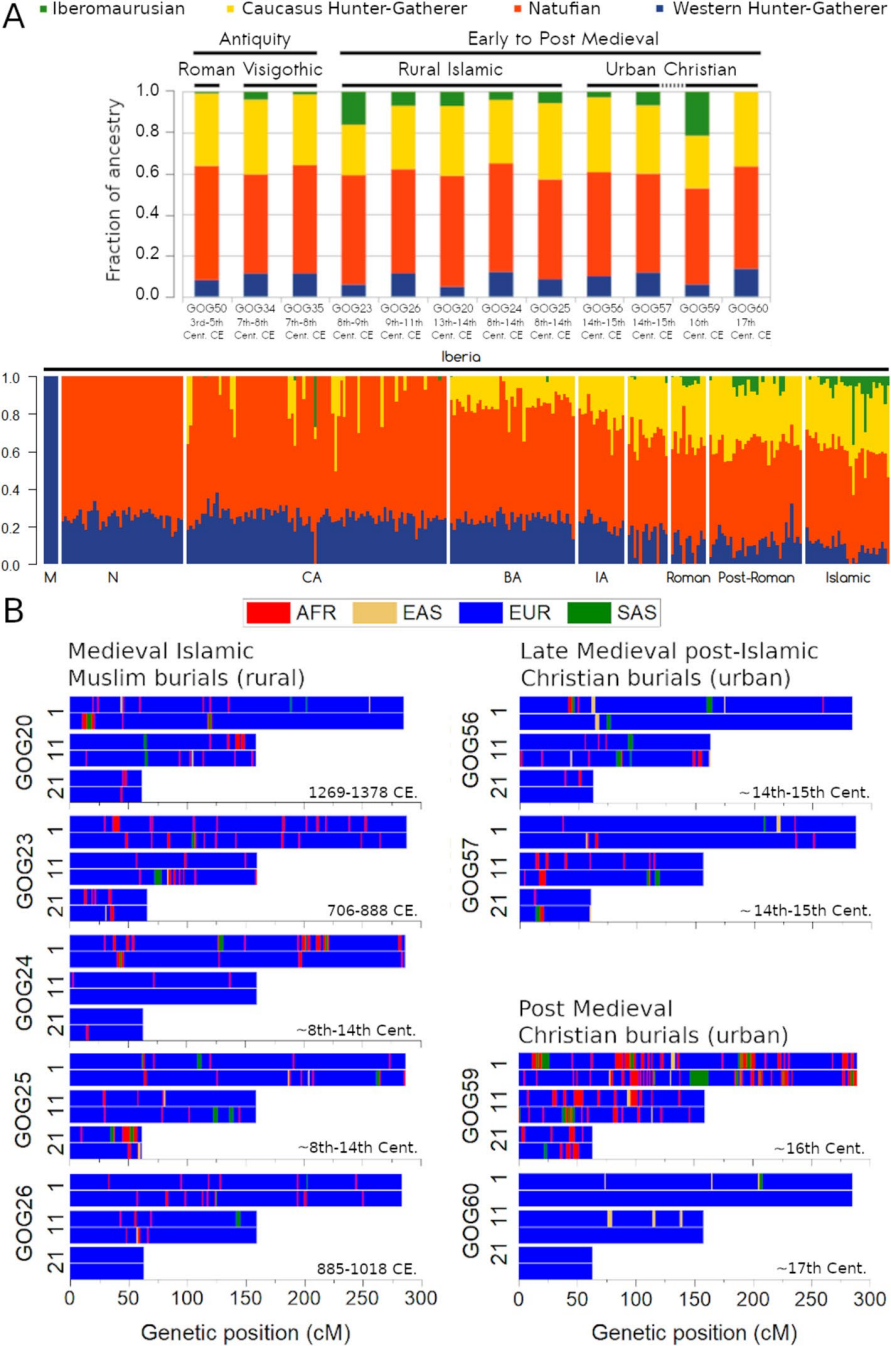
**A** Supervised ADMIXTURE (*K* = 4) defining a distal model, using as proxy sources for ancestry determination differentiated hunter-gatherer groups from Europe (WHG), the Caucasus (CHG), the Levant (Natufians), and North Africa (Iberomaurusians). **B** RFmix chromosome ancestry segments colored according to four possible sources from the 1000 Genomes Project: African (AFR) as proxy for North African contribution, European (EUR) representing the Iberian contribution, East Asian (EAS) and South Asian (SAS) as proxies for broad Asian ancestry

The two kindred individuals from late antiquity (GOG34 and GOG35, father and daughter, respectively) occupy a space with some other post-Roman and medieval Islamic samples from Iberia, also shifted toward western North African individuals on the North African cline of the PCA. ADMIXTURE and *qpAdm* results indicate that the father (GOG34) carried North African-related ancestry (tested using *Iberomaurusian* and *Morocco_EN* as proxies, respectively) (Fig. 3A; Additional file 2: Table S13 and S14). However, in ADMIXTURE, the daughter (GOG35) displays approximately half the amount of such ancestry than the father. This likely indicates that the mother contributed none and suggests different genomic backgrounds of the two parents. The case of GOG34 is interesting since his genome-wide pattern aligns with the core of Islamic genomes and his median C14 dating (734 cal. CE) makes him contemporary with the start of Islamic conquest—yet the archeological context appears pre-Islamic.

Supervised and unsupervised ADMIXTURE, *qpAdm* models (Fig. 3A; Additional file 1: Fig. S6; Additional file 2: Table S14) and LAI results (Fig. 3B; Additional file 1: Fig. S9) confirm that all pre-Islamic individuals carried North African ancestry, but at lower levels than the later medieval Islamic individuals. In pre-Islamic times, the influx of North African-related ancestry was more sporadic, since it is not found consistently across all published individuals [2]. It is only with the onset of the Islamic period that it becomes more sustained and consolidated at a higher proportion (Fig. 3A).

### Survival of widespread North African-related ancestry until the seventeenth century in the Valencian kingdom

The group of medieval Islamic individuals (GOG20, GOG23, GOG24, GOG25, GOG26) appears homogeneous in the PCA (Fig. 2A) with the exception of GOG23. They form a core cluster together with pre-Islamic GOG34 and two individuals (GOG56 and GOG57) from the post-Islamic late medieval period (Fig. 2A), in addition to some other published Islamic individuals from elsewhere in Spain [2]. We identify this cluster space as a consolidated “Berberized” population that formed largely during the Al-Andalus era (Fig. 1F; Additional file 1: Fig. S5 and S6). In our region of study, North African-related ancestry is present in these individuals at ~ 14–18% estimated with *qpAdm* (with SEs < 3% and *p*-values ranging from 0.43 to 0.89) (Additional file 2: Table S14). These are slightly higher levels than in the earlier individuals, as also seen in the supervised ADMIXTURE (Fig. 3A) with distal source populations. GOG23 is the oldest Islamic burial dated in our dataset (706–888 cal. CE) and constitutes, to our knowledge, one of the earliest examples of an Islamic burial from eastern Iberia to be studied genetically. This individual is drawn even closer in the direction of North African populations in the PCA (Fig. 2A) and had the highest amount of North African-related ancestry, as shown by the ADMIXTURE (Fig. 3A), *qpAdm* (~ 31.3 ± 2.5% (± 1 SE); *p* = 0.13), and LAI (Fig. 3A, 3). Using temporally proximal sources, we could model GOG23’s ancestry as deriving ~ 50% from a source genetically resembling *CanaryIslands_Guanche.SG* and ~ 50% from a Southern European source similar to either *Portugal_LateRoman.SG* (*p*-value = 0.09; SE = 4.9%) or *Italy_RomanImperial.SG* (*p*-value = 0.14; SE = 4.6%) (Additional file 2: Table S13).

The two late medieval samples (GOG56 and GOG57) from the fourteenth–fifteenth century CE were recovered from a Christian cemetery belonging to the parish of San Lorenzo in the city of Valencia. However, these two individuals still group within the medieval “Berberized” PCA cluster (Fig. 2A) two centuries after the Christian conquest of the city. The levels of North African-related ancestry are still comparable to those observed in the Islamic period (Fig. 3; Additional file 2: Table S14).

Post-medieval individual GOG59, who was found with an iron shackle around his right ankle, clusters closely with modern Moroccans and Algerians in the PCA (Fig. 2A), pointing to a North African origin. Most present-day Berber individuals in the North African PCA fall along a cline defined by the amount of sub-Saharan ancestry. GOG59 sits at the low sub-Saharan ancestry side of this cline (Additional file 1: Fig. S4 and S5). Outgroup-*f3* analysis shows that the highest shared drift is with present-day Moroccan Berbers (Fig. 2B). We can model much of his ancestry as deriving from a North African-related source (Additional file 2: Table S13 and S14). This result is further supported by a second PCA computed using an alternative Affymetrix North African dataset where GOG59 appears closest to present-day Berber individuals from Morocco and Algeria (Additional file 1: Fig. S4 and S5).

In contrast, the other post-medieval individual from the same cemetery, GOG60 (Fig. 2B), was the only one in our dataset that fully clustered in the PCA within the modern Spanish population (Fig. 2A). No traces of North African-related ancestry could be identified with ADMIXTURE or *qpAdm* analyses (Fig. 3A, Additional file 2: Table S13 and S14). The LAI profile (Fig. 3B) also indicated no discernible trace of sub-Saharan African haplotypes in the tested chromosomes. Furthermore, neither uniparental marker indicates affiliations to the Maghreb, but instead rather typical European lineages (Table 1). This profile is in sharp contrast with GOG59, who appears to be of full North African origin (when compared to a present-day dataset). It also contrasts with the immediately preceding individuals from the late medieval period (Figs. 2 and 3)—whether Muslim or Christian—and the three individuals from antiquity (Additional file 1: Fig. S9).

### Potential genomic impact of social isolation of Mudéjar and Morisco communities

Based on the information gathered in the analyses above, we combined some individuals into groups (GOG20, GOG24, GOG25, GOG26 as “Medieval Islamic” and GOG56 and GOG57 as “Late Medieval Christian”), while treating individually those plotting separately on the PCA (Fig. 2A) (GOG59, GOG60, and GOG23). Furthermore, by combining the dates (Fig. 1C; Additional file 1: Fig. S11), genetic ancestry, and burial context, we argue that we can identify some individuals as belonging to different socio-historical groups: GOG23 and GOG26 fall into the *Muladi* category (Christian converts to Islam), GOG20 into the *Mudéjar* (Muslims in Christian territories) and GOG56 and GOG57 could be identified as *Morisco* (Muslims forcibly converted to Christianity) or descendants thereof.

To measure the evolution of genetic affinities of our archeological samples with modern populations from Iberia and North Africa, we ran outgroup-*f3* tests by merging populations from the Human Origins and Arauna et al. [77] datasets (Fig. 2B). The results obtained conform to what we observe in the PCA, ADMIXTURE, *qpAdm*, and LAI results. The measurements of shared drift indicate a trend of increased affinity toward modern Iberian groups as the samples become more recent, with the striking exception of GOG59 (Fig. 2B). Both GOG23, who was an early Islamic individual with some Iberian-related ancestry, and GOG59, who seems fully North African in ancestry, displayed the highest outgroup-*f3* values with present-day Maghrebi populations, especially Moroccan Berbers from Tiznit (Morocco_TIZ_BER) (Fig. 2B). The Islamic core group of four individuals (GOG20, GOG24, GOG25, GOG26) do not show particularly high affinities with either North African/Berber groups or Iberians/Europeans, highlighting more balanced contributions of both sources to their ancestry (Fig. 2B) in relation to the other samples analyzed. The late medieval Christian grouping showed a similar trend, although with signs of increased affinity with present-day Basques (Fig. 2B). Finally, GOG60, the post-medieval individual from the seventeenth century, displayed the opposite pattern to GOG59, with the highest *f3* values shared with Iberian groups (Fig. 2B).

Due to coverage and sample size constraints, in order to study ROH patterns in depth we combined two approaches: hapROH on the whole genome based on pseudo-haploid genotypes and oROH/pROH (see “Methods”) for three imputed chromosomes. The use of hapROH [78] allowed us to explore all chromosomes (although focussing only on a subset of 1240k sites) and integrate previously published 1240k capture data, but with loss of individuals from our transect. With imputation, we evaluated all individuals in our transect, but in a subset of chromosomes. We validated the concordance of both approaches through the identification of the same ROH fragment in chromosome 21 of individual GOG20 (Additional file 1: Fig. S7 and S12).

**Fig. 4:**
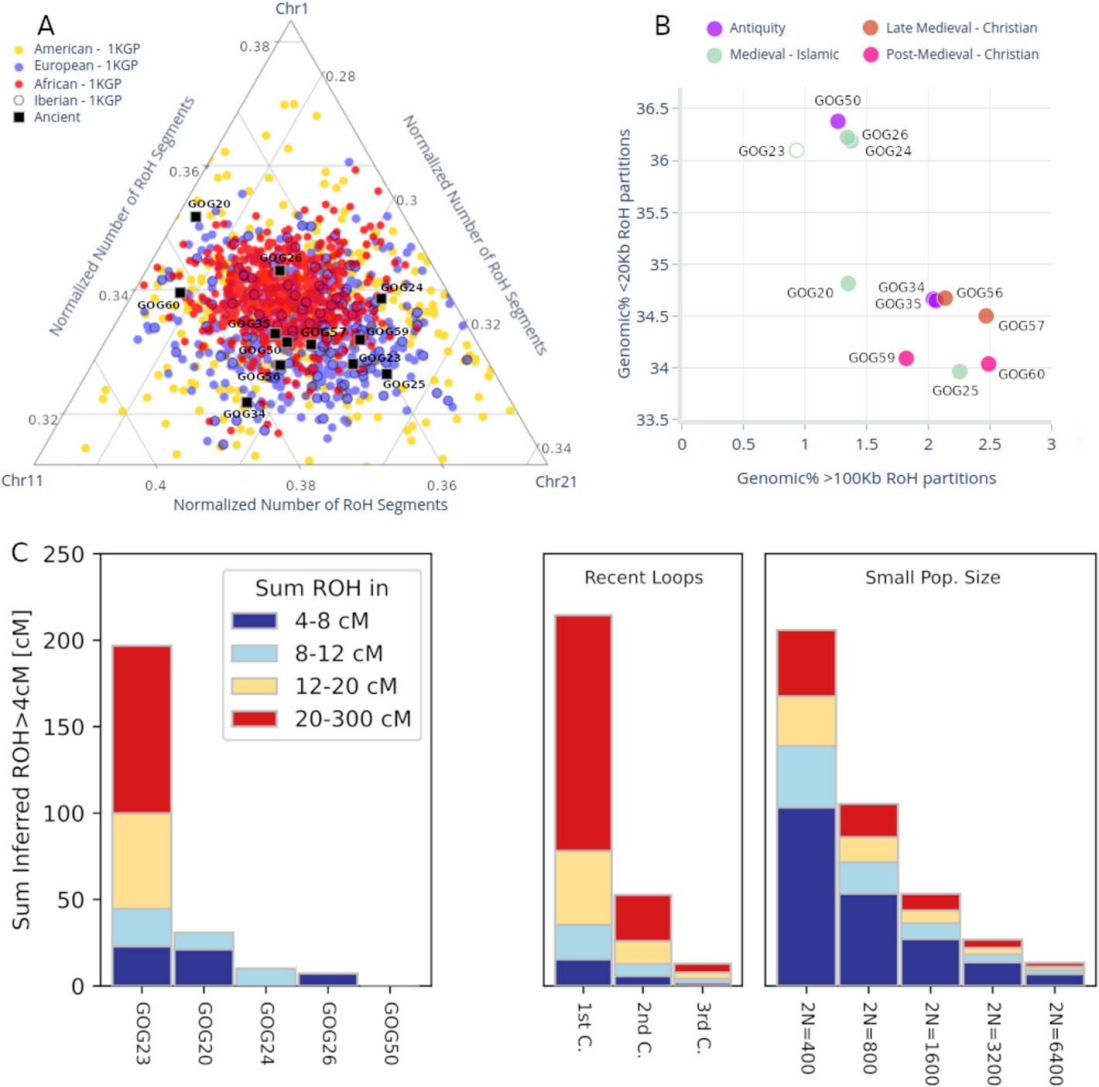
**A **Number of runs of homozygosity identified by PLINK (pROH) and normalized by the length of each chromosome. **B **Genomic fraction across the three imputed chromosomes covered by short (< 20 kb) and long (> 100 kb) segments of organic ROH (oROH) in a large (Chr1), medium (Chr11), and small (Chr21) chromosome. GOG23 is unfilled to denote its uncertainty. **C** hapROH results for the viable subset of the new samples reported in this work

In the context of modern worldwide populations, none of the ancient individuals analyzed seemed to suffer from extreme levels of consanguinity based on the number of pROH fragments found in the imputed chromosomes (Fig. 4A). Three of them (GOG20, GOG34, GOG60) appear on the fringes of typical European variability (Fig. 4A). However, hapROH identified that the early Islamic burial (GOG23) carried a combined total of ~ 200 cM in ROH > 4 cM, with a high proportion of very long ROHs (> 20 cM), consistent with being the offspring of closely related relatives (Fig. 4C; Additional file 1: Fig. S12); a signal missed with oROH since the ROH segments fall in different chromosomes (Additional file 1: Fig. S12).

Bearing this caveat in mind, we attempted to explore the patterns in finer detail. A deeper look within the set of ancient genomes analyzed here suggests increased inbreeding over time in the medieval population of Valencia (Fig. 4B). Using the oROH from the imputed chromosomes as reference (Fig. 4B), the Roman individual (GOG50) together with the two Islamic genomes (GOG24 and GOG26) appear to have the highest percentage of the genome in short organic ROH (20 Kb > 35%) and the lowest of long organic ROH (100 kb < 1.5%) (Fig. 4B). This group also included GOG23, but we excluded him from this inference, since hapROH revealed high inbreeding in the rest of his chromosomes (Fig. 4C).

The remaining group of medieval individuals are more recent burials, except for GOG25, whose exact age is unknown. In this second group, samples exhibited lower percentages of short oROH (< 35%) and a higher fraction of long oROH (> 1.5%) (Fig. 4B). The only exception was GOG20, the Islamic individual with the most recent radiocarbon date (1285 cal. CE. median age) whose fraction of long ROH was also below 1.5%. The two kindred individuals from late antiquity (GOG34 and GOG35) are more akin to each other and group with the later Islamic and medieval samples, rather than with the late Roman female individual (GOG50).

## Discussion

The results presented here buttress the idea that gene flow from North Africa into Iberia was ongoing in the centuries preceding the Islamic period. However, we also show that this gene flow was not restricted to southern Iberia [2], but it impacted eastern Iberia since at least late Roman times. This inflow is evidenced by the finding of North African ancestry in all pre-Islamic genomes, albeit at low levels. Our data also suggest that, during the centuries of Roman Imperial rule, there was a significant dynamic of pan-Mediterranean homogenization contributing sporadic Asian-related ancestry, as exemplified by individual GOG50. With lesser intensity, this mirrors the dynamic observed in Rome itself [6], a phenomenon most likely driven by mobile peoples from Italy, Greece, Asia Minor, and the Eastern Provinces as slaves or otherwise. However, more genomes are necessary for a full comparison with other localities. This transient phenomenon hints at a much more complex picture if we wish to understand and quantify accurately the genetic contribution of North African migrants in the later Islamic era. We also highlight the apparent lack of ancestry contribution from native peoples from the Arabian Peninsula during the Al-Andalus epoch (Fig. 1F) in the genomes studied in this work.

Note that a shortcoming of studies focusing on the Islamic period is the lack of contemporary North African and Arabic ancient genomes—a gap that should be filled in the future.

The profile of the genome recovered from the burial of the female (GOG50) from the Roman colony of Valencia provides interesting insight into the heterogeneity of the Roman population of the city. The local Iberian population in coastal regions may have been no stranger to North African influences, and the existence of Romance languages in North Africa could have been a factor aiding the later swift Islamic conquest [79]. This is supported by the fact that the two post-Roman individuals also carried North African-related ancestry. The Islamic conquest of the Iberian Peninsula, beginning in 711 CE, further intensified this phenomenon by intertwining the Maghreb and parts of Iberia intimately together [45], also seen at the genetic level [15].

The genomes from the Islamic period therefore point to an intensification and consolidation of the genetic “Berberization” in eastern Iberia during the Middle Ages, rather than a completely new phenomenon. The genetic structure of medieval Iberia became a patchwork resulting from the shifting religious and cultural geographies.

We argue that the Islamic burials who carried a higher North African contribution in their genomes were likely representatives of the *Muladi* population: people of Iberian descent who adopted Islamic culture and religion and inter-married (the original meaning of *muladi* in Arabic was a person of mixed Arab and non-Arab ancestry [80–82]). The oldest Islamic individual presented here, GOG23, lived within the first generations following the conquest, and had North African and an Iberian-related ancestries, suggesting that acculturation was relatively swift and accompanied by admixture between locals and newcomers. However, his high level of consanguinity might suggest a limited mating pool conducive to close-kin marriages. A similar hypothesis was also suggested for other close-kin offspring in seventh–eleventh century CE Iberian sites, both in Islamic and Christian contexts [18, 83], but may not be applicable in the case of a previously published individual (I7457) from a later site in Granada dating to the Almohade period (Additional file 1: Fig. S10B) [2]. Refined dating and more ancient genomes at higher coverages, as well as contemporary genomes, would be necessary to identify any possible temporal and/or geographical trends in ancestry, consanguinity, and effective population size in Islamic burials spanning key transitional periods.

On the other hand, the two late medieval Christian burials from Valencia, who carried similar fractions of North African-related ancestry, may be representatives of the *Moriscos*, the people who converted to Christianity at the beginning of the sixteenth century CE. Mass conversions would explain the obvious genetic continuity, despite the alleged Muslim population displacements and socio-political changes experienced following the Christian conquest.

The apparent trend of growing homozygosity through the medieval period—genomic profiles with similar ancestry composition have more longer organic ROH and fewer short ones in early medieval individuals than in later medieval samples—could be the product of “habitat fragmentation” by means of loss of connectivity, social isolation, and reduction of *Mudéjar* and *Morisco* communities in the new Christian society. We caution that this observation can only be speculative given the small sample size and should be tested when more radiocarbon dates and genomes become available. Regarding the post-medieval individual GOG60, the reasons for his levels of homozygosity must be found elsewhere. He might originate from another isolated social group of Christians in Islamic society like the *Mozarabs*. Our results also shed light on urban dynamics in Valencia. Despite property seizure by the new Christian elites [47, 82], historically described expulsion of native Muslim citizens, and re-population with Aragonese and Catalan people, the genetic make-up inside the cemetery of San Lorenzo (Valencia) did not change compared to the preceding rural Islamic population from Vall d’Uixo and with other urban Islamic individuals from Valencia [2, 15].

It is striking that our sole seventeenth century genome (GOG60) is the only individual lacking North African-related ancestry. Although it is only a single sample, this result is consistent with the lower fraction of North African ancestry found in the modern population of the Valencian territory [3, 19, 84]. We provisionally interpret this pattern as a reflection of a historical event that occurred in 1609 CE: the Expulsion of the Moriscos from the Kingdom of Valencia. This expulsion, and repopulation with peoples from northern territories, effectively erased the local North African component. Christians, especially those from more northern regions, likely carried less North African-related ancestry than the Moriscos that they replaced [2, 15], although this remains to be tested. It must be noted that individuals without North African ancestry, such as GOG60, may have also lived in Valencia before the seventeenth century.

On the other hand, the presence of an enslaved male individual (GOG59) of Berber origin, buried in a Christian cemetery, highlights the lower status of inhabitants with links to the Barbary Coast following the *Reconquista* and before the final expulsion. The elucidation of the genetic origin of GOG59 offers an insight into the provenance of some of the slaves during the Valencian Golden Age (fourteenth to sixteenth centuries CE), and substantiates historical accounts of Christian raids to capture and enslave people in North Africa [85], although the provenance of slaves was not limited to North Africa [86–88].

One final point, highlighted by the survival of North African-related ancestry in substantial proportions until the seventeenth century, is the widespread presence of such ancestry in present-day South Americans [89]. Christian converts were forbidden to migrate to the Americas, although clandestine journeys probably occurred. However, the North African-related ancestry signature seen today in South America is too high to be satisfactorily explained by sporadic movement. The high estimates of North African ancestry in South America suggests that colonial migration involved people carrying higher levels of this ancestry than the average in present-day Spain [3, 89]. Furthermore, the time estimates since the North African admixture in South America are consistent with the Iberian admixture episode [89]. This strongly suggests that most of this ancestry was introduced by the initial colonial immigrants. The two late medieval individuals from Valencia further support this observation: a population with increased North African-related ancestry existed at the time in Spain, likely not only in Valencia. Given that cities in the south, such as Sevilla and Cádiz, were the main ports for the colonial voyages to America, we hypothesize that North African-related ancestry also survived in southern regions after the end of the Islamic period and became the source of such ancestry introduced in South America.

## Conclusion

Our aDNA results suggest little to no effect of the so-called *Reconquista* in the genomic ancestry of the medieval Valencian population. We observe that the Islamic rural population is not different from late medieval Christians in an urban context. Instead, we find evidence of a much later shift in the genetic composition of the Valencian population, as a direct result of a post-medieval episode of ethnic cleansing and cultural genocide early in the seventeenth century: the Expulsion of the Moriscos. This was followed by a repopulation effort with Aragonese, Catalan, and Navarrese migrants. Under Philip III, an estimated 300,000 *Morisco*s, or a third of the population, were expelled to North Africa from across Spain—a much larger figure than even the Jewish expulsions in 1492 CE. These people, with a mix of Iberian- and substantial North African-related ancestries, had been the inhabitants of the region for hundreds of years and had been forcibly converted from Islam to Catholicism a century earlier following the *Reconquista*. Despite a hugely civilizing legacy on the culture of Europe, particularly of the Iberian Peninsula, that endures to the present day, most of the last visible vestiges of the *Moors* of Spain were largely dispersed across the Mediterranean between 1609 and 1614 CE. As a result, a genetic bridge between Europe and Africa that had been in place for centuries, and whose legacy we can detect in the genomes of those living in the Valencian region up until the seventeenth century, was thoroughly dismantled.

## Methods

### Sampling and sequencing

We handled and processed archeological samples for aDNA extraction, in a dedicated aDNA facility at the University of Huddersfield. We processed a total of 35 samples, comprising a range of different skeletal elements: petrous bones, molars, phalanxes, and a metatarsus. We followed established protocols specific for DNA extraction from ancient remains [90–92]. Library preparation followed the Meyer et al. [93] protocol with modifications [94, 95].

We screened 35 double-stranded (ds) DNA libraries (without post-mortem damage/USER™ treatment), one per sample, on the Illumina MiSeq platform at Trinity College Dublin to evaluate endogenous DNA content (Additional file 1: Fig. S1). We confirmed aDNA post-mortem damage patterns in the screening non-USER™-treated libraries with mapDamage (v.2.0.7) [96]. We then selected the most promising samples and generated USER™-treated dsDNA libraries for further paired-end sequencing on the Illumina HiSeq 4000 platform at Macrogen (Seoul, South Korea).

### Data processing

We evaluated paired-end read quality with FastQC (v.0.11.5) [97], removed adapters and merged paired-end sequencing reads using leeHom [98] with the flag –ancientdna. We mapped reads against the human genome reference hg19 with rCRS as mitochondrial reference using BWA aln and samse commands (v.0.7.5) with specifications (-l -n 0.01, -o 2) [99]. We performed quality control of the resulting BAM files with QualiMap (v.2.262) [100]. We removed duplicated reads using the rmdup command in Samtools (v.1.16) [101]. To avoid SNP miscalls due to post-mortem damage, we soft-clipped 3 base pairs at the ends of the reads using the trimBam option in BamUtil package (v. 1.0.14) [102]. We filtered out reads shorter than 34 bp in length and under mapping quality 20. We added read groups using Picard Tools [103] before merging libraries. Finally, for samples with more than one sequenced library, we merged BAM files that had been treated independently up to this point, using the merging option in Picard.

### Uniparental marker classification and kinship determination

We mapped mitochondrial reads to mitochondrial sequence NC_012920.1 (rCRS). We classified haplogroups using HaploGrep 2.0 [104, 105] and curated them following the nomenclature in PhyloTree (Build 17, February 2016) [106]. We classified haplogroups for Y chromosomes in male samples using Yleaf [107] following ISOGG 2019 nomenclature [108]. We also assigned Y-chromosome haplogroups with pathPhynder [109]. We inferred biological kinship relationships with READ [110].

### Population genetic analyses

We used Samtools mpileup with minimum base and mapping quality of 20 to make SNP calls and pileupCaller [111] to select one allele at random to create pseudo-haploid genotypes overlapping with the HO (for PCA) and 1240k SNP panels (other analyses). We used the non-pruned dataset for PCA and pruned for ADMIXTURE and *f*-statistics. We used EIGENSOFT [112, 113] functions *convertf* and *mergeit* to convert and merge files when necessary and computed PCAs using a least squares projection of ancient samples onto modern populations using smartpca (lsqproject: YES, shrinkmode: NO). We ran *qpAdm* using different formulations to test a variety of distal and more proximal models of ancestry. We filtered SNPs for linkage disequilibrium (LD) using the command –indep-pairwise (200, 25, 0.4) in PLINK1.9 [114] for *f*-statistics (computed using qp3Pop and qpDstat in the ADMIXTOOLS package [115]) and ADMIXTURE [116]. We provide additional details on the datasets and parameters used in the supplementary material.

### Runs of homozygosity on pseudo-haploid data

We estimated runs of homozygosity (ROH) longer than 4 cM using hapROH (with default settings), optimized for low-coverage pseudo-haploid aDNA data [117]. We restricted the analysis to individuals with at least 400,000 SNPs overlapping with the 1240k SNP panel: GOG20, GOG23, GOG24, GOG26, from medieval/Islamic contexts, and GOG50, dated to the Roman period, as well as previously published Mediterranean individuals dating to the last 2500 years [2, 6–8].

### Simulation of hybrid genomes

We simulated hybrid genotypes resulting from a source of present-day Moroccan individuals and another from present-day individuals from Spain (as reported in the HO dataset), similarly to Haber et al. [5]. We projected the simulated hybrid genomes onto a PCA calculated on a merged HO/Arauna et al. [77] dataset with ~ 20 k overlapping SNPs.

### Imputation and local ancestry inference (LAI) and organic homozygosity

For imputation of chromosomes 1, 11, and 21, we first generated VCF files containing all the variants in each chromosome for each individual using GATK UnifiedGenotyper [118]. We combined BEAGLE 4.0 and BEAGLE 5 [119] to phase and impute the missing genotypes, as detailed in Hui et al. [120], making use of the 1000 Genomes (1 KGP) Phase 3 reference dataset [121].

We used the VCF files to obtain LAI profiles of the three imputed chromosomes. LAI was carried out with RFMix v2 [122] with default parameters. As reference haplotypes, we used the phased VCFs from the 1000 Genomes Project together with the corresponding genetic maps.

Since hapROH could not be applied to all individuals, we complemented hapROH by using the imputed chromosomes VCF files to calculate organic runs of homozygosity (oROH) and heterozygosity. We also calculated ROH statistics using PLINK (pROH) with default parameters.

## Supplementary Information

Additional file 1: Supplementary information about the archeological sites, methods, figures and tables list.

Additional file 2: Supplementary tables S1-S15 contents.

Additional file 3: Raw data at ENA information.

## Data Availability

Data available at the European Nucleotide Archive (ENA) database under the accession number PRJEB65253 [123].
