# Supplementary material for: Medieval genomes from eastern Iberia illuminate the role of Morisco mass deportations in dismantling a long-standing genetic bridge with North Africa
Source: Genome Biol. 2025 Apr 28;26:108. doi: 10.1186/s13059-025-03570-1 (PMC12036142; doi:10.1186/s13059-025-03570-1)
Supplement: Supplementary file 1 — Additional file 1: Supplementary information about the archeological sites, methods, figures and tables list. [file 13059_2025_3570_MOESM1_ESM.docx]

**SUPPLEMENTARY INFORMATION**

Medieval genomes from eastern Iberia illuminate the role of Morisco mass deportations in dismantling a long-standing genetic bridge with North Africa

Gonzalo Oteo-García*^1,2,3^, Marina Silva^1,4^, M. George B. Foody^1^, Bobby Yau^1^, Alessandro Fichera^1^, Llorenç Alapont^5^, Pierre Justeau^1^, Simão Rodrigues^1^, Rita Monteiro^1^, Francesca Gandini^1^, María Luisa Rovira Gomar^6^, Albert Ribera i Lacomba^7^, Josep Pascual Beneyto^8^, Valeria Mattiangeli^9^, Daniel G. Bradley^9^, Ceiridwen J. Edwards^1^, Maria Pala^1^, and Martin B. Richards*^1^

^1^School of Applied Sciences, University of Huddersfield, Queensgate, Huddersfield, HD1 3DH, UK

^2^Dipartamento di Biologia Ambientale, Sapienza Università di Roma, Rome, Italy

^3^Centre for Palaeogenetics & Department of Archaeology and Classical Studies, Stockholm University, Stockholm, Sweden

^4^Ancient Genomics Laboratory, The Francis Crick Institute, London, UK

^5^Department of Prehistory, Archaeology and Ancient History, University of Valencia, Valencia, Spain

^6^Museu Arqueològic Municipal de la Vall d’Uixó, Vall d’Uixó, Spain

^7^Centro Arqueológico de l'Almoina & SIAM, Valencia, Spain

^8^Museu Arqueològic d’Ontinyent i la Vall d’Albaida, Ontinyent, Valencia

^9^Smurfit Institute of Genetics, University of Dublin, Trinity College, Dublin, Ireland

Corresponding authors: Gonzalo Oteo-García ([gonzalo.oteogarcia@uniroma1.it](mailto:gonzalo.oteogarcia@uniroma1.it); [gonzalo.oteo@su.se](mailto:gonzalo.oteo@su.se)), Martin B. Richards ([m.b.richards@hud.ac.uk](mailto:m.b.richards@hud.ac.uk))

**Table of contents:**

- Supplementary Material and Methods
- Supplementary Tables list
- Supplementary Figures
- Supplementary References

**Supplementary Materials and Methods**

**Sampling locations**

Once permissions to access the samples were obtained, sampling for the following locations was arranged to be carried out. Following screening for the content of endogenous DNA, the best preserved samples were selected for further sequencing. These twelve individuals were drawn as the best from the total 35 screened samples which had yielded endogenous DNA proportions ranging from 0.01% to 41.58%. The average endogenous DNA content was about 7%. The sequencing of the DNA recovered from bones included petrous bones, molars and a metatarsus, and yielded average nuclear coverages, after mapping quality filters, ranging from 0.21× to 2.34×.

• Necropolis de La Union (Vall d’Uixó): cemetery from the Visigothic period with over 40 inhumations (39.816157, -0.234099). This site is peculiar since almost all the graves had several individuals buried together. The archaeological interpretation is that the individuals in shared graves share some familial link. They all appear to be victims of a violent event, and they died within a short period of time. Many individuals display signs of violence. We sampled five individuals from this site but none yielded enough endogenous DNA contents to be sequenced further. No radiocarbon dates available. Samples: GOG15, GOG16, GOG17, GOG18, GOG19 (1).

• Islamic Maqbaras (Vall d’Uixó): these are a series of burials discovered during various excavations in the 1990s across Vall d’Uixó. They have been identified as the Islamic cemeteries of agricultural hamlets or farms (Alquería Benigafull (39.823088, -0.235724), Alquería Benizahat (39.824500, -0.229082), and Alquería Ceneja (39.822567, -0.23749) that formed the primitive cores of the Vall d’Uixo settlement. The locations of Alquería Ceneja and Alquería Benifagull were on the high ground of the modern town. Alquería Benizahat was on the lower part of the modern town. These settlements eventually fused to originate the modern municipality (2). Archaeological inferences estimated that the settlements on the high ground are older than the hamlets in the lower ground. We sampled nine individuals from four of these sites. The samples were initially believed to be from between the 11th and 14th centuries. However, the radiocarbon dating performed on three of the samples revealed a wider time frame, from the 8th to the 14th century. Samples: GOG20, GOG21, GOG22, GOG23, GOG24, GOG25, GOG26, GOG29, GOG30 (1).

• Sanxo Llop and La Vital (Gandia): two excavation sites in very close proximity to one another. La Vital (38.969286, -0.167254) is an archeological site with evidence of human occupation going back to the Late Neolithic. From this location we collected three samples from an early medieval pre-Islamic context, but endogenous DNA contents were insufficient for further sequencing and these samples were not included in further analyses. In Sanxo Llop (38.963488, -0.169599), excavations revealed a burial ground dating to late antiquity, overlapping between the late Visigothic period and early Islamic decades, at least between 600 CE and 800 CE according to available radiocarbon dates. We sampled two individuals (an adult and a sub-adult) that revealed good preservation of endogenous DNA. The two individuals were deposited in a round pit that had likely been used for other purposes in the past. Samples: GOG31, GOG32, GOG33, GOG34, GOG35 (1,3).

• Cementerio de San Lorenzo (Valencia): Chrisitian cemetery inside the walled perimeter of medieval Valencia (39.477944, -0.375865). In use since at least the 15th century up until the mid 19th century. In the 19th century the growing consensus that cemeteries inside cities were a health hazard led to the development of burial grounds outside urban areas. In 1841 following the confiscation of urban cemeteries, all the known and recent graves were moved to the newly built Cementerio General de Valencia. Excavations in 1999 on the site of the former cemetery at Calle Sabaters & Plaza Cisneros found the older non-translocated medieval graves, spanning from the 14th century to the 17th. The same area covered by the medieval cemetery had been used in Late Roman times for burials and the excavation found the remains of two of these Roman tombs. We sampled eight individuals from the 14–17th century period and the two individuals from the Roman graves. The remains from the Roman burials were poorly preserved but one yielded exceptionally high amounts of endogenous DNA. Samples: GOG49, GOG50, GOG53, GOG54, GOG55, GOG56, GOG57, GOG58, GOG59, GOG60 (1).

• Other locations: we sourced other bone material that was unsuccessful in the recovery of ancient DNA (aDNA). These other samples were scattered an include one Islamic burial (GOG45) from an excavation in Calle Ripalda (Valencia), three Islamic samples (GOG13, GOG14, GOG46) from Plaza del Almudín in Segorbe (a site previously studied in (4)) and two medieval samples (GOG51, GOG52) from Monasterio San Vicente de la Roqueta (Valencia) (1).

**Processing of ancient bones for aDNA**

The handling of ancient DNA material was carried out at the dedicated Ancient DNA Facility run by the Archaeogenetics Research Group at the University of Huddersfield. The Ancient DNA Facility is isolated from other molecular biology facilities handling post-PCR products or modern sources of DNA since it is located in a separate building of the Queensgate Campus (Huddersfield). In every work session, handlers were outfitted with a full-body Tyvek suit, a hairnet and face mask and a double layer of gloves at all stages. The facility includes two independent rooms dedicated to sample drilling and DNA extraction/library preparation respectively. The protocol required all materials and surfaces were regularly cleaned with LookOut® DNA Erase (Sigma-Aldrich), bleach and by exposure to UV-radiation after each session. To reduce risk of bacterial and other contamination, the samples selected to be processed by drilling were UV-radiated for a total of 60 minutes (30 minutes for each side) before being carried to the sample drilling room. We cleaned the sampling surfaces of the petrous bones or molars by air-abrasion, with 29 µm aluminium oxide powder (OEA Labs) and a SWAM-Blaster® compressed air abrasive system (Crystal Mark). We used a Micromotor System Maxima drill with a 22 mm diameter diamond cutting edge for sampling. For teeth we separated the root and crown to powder the roots for DNA extraction. For petrous bones, we extracted a bone wedge from the densest part (5). In the case of other bones (phalanx and tarsus) we targeted the epiphysis. Once a piece was obtained, we powdered the samples using a Mixer Mill (Retsch MM400) for 45 seconds at a frequency of 30 Hz/s.

**Radiocarbon dating**

We dated petrous bones from four individuals (GOG20, GOG23 and GOG26 from La Vall d’Uixo, and GOG50 from the Cementerio San Lorenzo) at 14Chrono, the radiocarbon dating lab at Queen’s University (Belfast). We calibrated the resulting dates in calibrated (cal.) CE using OxCal version 4.4.4 and the most recent calibration curve, IntCal20 (6,7). Sample GOG34 had been dated in the past at Beta Analytic (Miami) by means of AMS measurements made with NEC SSAMS accelerator mass spectrometers (Figure S12).

**Extraction of aDNA**

We followed a modified protocol extraction developed for DNA from ancient skeletal remains (8–10). The extraction buffer (EB) contained 20 mM of Tris HCL, pH 8; 50 mM of EDTA, pH 8, RNase and proteinase free, and 0.5% of SDS (DNase, RNase and protease free, heated to a temperature of 37ºC). All components were exposed to UV light for 15 minutes before addition of proteinase K. We performed a two-step digestion: bone or tooth powder were incubated rotating with 1 mL of extraction buffer for approximately 24 hours at 37 ºC, followed by centrifuging at 13,000 rpm for 15 minutes to separate mineral particles from the supernatant (which was retained at -20ºC). After the second incubation the tubes were again centrifuged at 13,000 rpm again for 15 minutes and the supernatant was transferred to 6 mL Corning® Spin-X® UF Concentrator tubes, to which we added 3mL of 10mM Tris HCL (pH 8) and centrifuged for 20 minutes at 2,500 rpm twice (the flow-through was discarded after the first centrifugation, and 3mL of 10mM Tris HCL (pH 8) added again). We retained a final volume of ~100 µL, which was transferred to new silica columns (MinElute® PCR Purification Kit, commercialised by Qiagen) for purification following standard protocol by the manufacturer, plus addition of 0.05% Tween-20 (0.03 µL per sample) to 59.97 µL per sample of EB Buffer to reduce absorption of DNA to plastics and keep long-term viability of DNA extracts. The final 100 µL volume of DNA extracted was frozen. We confirmed DNA extraction with DNA quantification with a QubitTM 3.0 Fluorometer (ThermoFisher Scientific), using the Qubit® dsDNA HS Assay Kit (Invitrogen).

**Library preparation and sequencing**

The protocol from Meyer *et al.* (11), with modifications introduced in Gamba *et al.* and Cassidy *et al.* (12,13), was followed to make the library preparations for the samples. In between all main stages (i, ii, iii, iv and v) of library preparation, clean-up steps were performed using the MinElute PCR Purification Kit, according to manufacturer instructions, and adding Tween 0.05% to EB Buffer to obtain EBT Buffer in the same way as detailed above.

After an initial screening using a Illumina MiSeq platform (performed at the Trinity Genome Sequencing Laboratory, Trinity College Dublin, Ireland), the libraries with sufficient endogenous DNA (5%) for further sequencing were UDG-treated before library-preparation as follows: addition of 5.0 µL of USER® enzyme (Uracil-Specific Excision Reagent by New England BioLabs®) to 16.5 µL of DNA extract and incubated for 3 hours at 37ºC in order to remove uracil residues derived from post-mortem damage characteristic of ancient DNA (14–17) .

The double-stranded DNA library preparation protocol consisted of various stages which included blunt-end repair (i), adapter ligation (ii), followed by an adapter fill-in reaction with *Bst* DNA polymerase (iii). Indexing oligo sequences were added by amplification with IS4 primer (iv). Finally libraries to be sequenced together in the same lane were pooled together (v).

Step i) For each sample, we merged the cleaned DNA volume of 21.5 µL resulting from the USER-treatment, together with 3.5 µL of NEBNext End Prep Enzyme Mix, 7 µL of 10X of NEBNext End Repair Reaction Buffer (both included in the NEBNext® End Repair Module, New England BioLabs®), and 38 µL of ddH2O (sterile ultrapure water). The final volume (for one sample) was 70.0 µL that was later incubated at 25ºC for 15 minutes, followed by 5 minutes at 12ºC, and purified with the MinElute PCR Purification Kit.

Step ii) The adapter mixes of P5 and P7 (20 µM each) (by Sigma-Aldrich) were pooled together with 1 µL of T4 DNA ligase I (5U/µL), 10µL of ddH_2_O (sterile ultrapure water), 4 µL of 10X T4 DNA ligase buffer by Thermo Scientific, and 4 µL 50% PEG-4000 (Thermo Scientific). The final volume was 20 µL per sample and was pooled together with another 20 µL of eluate from the previous Step i, then it was incubated at 22ºC for 30 minutes. In this step, adapters were ligated by the activity of T4 DNA ligase catalysis of phosphodiester bonds between 5’- and 3’-ends in dsDNA.

Step iii) For the adapter fill-in step, we mixed 20 µL of DNA from Step ii with 13.5 µL of ddH_2_O (sterile ultrapure water), 4 µL ThermoPol® Reaction Buffer 10X, 1 µL of dNTP (10mM each), and 1.5 µL of *Bst* DNA polymerase (Large Fragment, 8U/µL), resulting in a total volume of 40 µL to be incubated for 30 minutes at 37ºC, followed by an extra 20 minutes at 80 ºC necessary to inactivate the *Bst* DNA polymerase and terminate the reaction.

Step iv) Library amplification reactions were prepared in the Ancient DNA clean room and the final amplification step was carried out in the post-PCR lab space. The reaction consisted of 41 µL of Accuprime *Pfx* SuperMix (Thermo Scientific), 1 µL of primer IS4 (10 µM), 2 µL of appropriate indexing oligo (both made by Sigma-Aldrich) plus 6 µL of sample library from Step iii. Total reaction volume is 50µL. The PCR reaction protocol consisted of an initial denaturation phase at 95ºC for 5 minutes, followed by 12 cycles of denaturation at 95ºC for 15 seconds, annealing at 60ºC for 30 seconds, extension at 68ºC for 30 seconds, and a final extension at 68ºC for 5 minutes. Finally, we performed the last clean-up step with the MinElute PCR Purification Kit.

Step v) We measured the concentration of the libraries with a Qubit 3.0 Fluorometer, using the Qubit® dsDNA HS Assay Kit and checked the fragment size distribution with a Bioanalyzer (Agilent), using the Agilent High Sensitivity DNA Kit. Libraries were evaluated visually and if deemed successful (majority of fragment lengths in the libraries peaking at 150–200 bp), libraries were pooled together. We sent the libraries to Macrogen (Seoul, South Korea) for whole genome next-generation sequencing (NGS) in Illumina HiSeq 4000 platforms.

**Processing next-generation sequencing data**

The paired-end raw FASTQ files were evaluated using FastQC (version 0.11.7 by Babraham Bioinformatics) to check for quality of the high throughput sequence data. Results of the checks were inspected visually. Once we cleared the FASTQ viability we proceeded to remove adapter sequences and merged paired-end files. We used leeHom (18) with the flag --ancientdna to merge paired-end reads and to remove adapters.

**Mapping next-generation sequencing data**

We used Burrows Wheeler Aligner (BWA, v.0.7.5a-r40560) with the commands samse and aln to generate BAM files mapped to the Human Reference Build 37 (hg19/GRCh37.p13). Ancient DNA specifications were used to disable the minimum seed length (-l option) and allow the aligning algorithm to be more flexible and map more reads increasing coverage (-n 0.01, -o 2). We used Qualimap (19) to retrieve alignment metrics for quality control.

We removed duplicates from the BAM files with Samtools (v1.15) rmdup. Once the duplicates were removed, quality filters were applied. A minimum mapping quality of 20 was chosen to reduce reference bias. Minimum read lengths were set at 34 base pairs long (13,20). DNA damage patterns were evaluated visually by looking at the plots generated with mapDamage (v.2.0.7). We soft-clipped three base pairs at the end of each read using the trimBam (–clip) function in bamUtil to remove the vast majority of the deamination damage, given that the sequences are already uracil–DNA–glycosylase (UDG) treated (21).

We added read groups at library-level using Picard Tools, and we merged all libraries from the same sample (which had been treated independently until now) into one file using the merging option in Picard.

**Data authenticity**

Anti-contamination measures were in place while drilling, extracting aDNA and during library preparation in the Ancient DNA Facility, as explained above. Multiple negative controls were also introduced in the chain leading to library preparation and sequencing. DNA quantification and sequencing results of these controls showed that the levels of contamination by exogenous DNA were negligible. We further checked authenticity of the data by checking the patterns of post-mortem damage and DNA fragmentation with MapDamage v.2.0.7 and BamDamage (22,23).

All non-USER-treated libraries generated for screening samples presented the typical misincorporation patterns of ancient DNA, which were also detectable in very low levels in the USER-treated libraries (Figure S2). We also checked levels of mtDNA contamination in the samples with Schmutzi. We further evaluated that the mitochondrial haplotypes of each individual were consistent with one haplogroup only.

**Genetic sex determination**

Genetic sex determination was established with Ry score (24) on all individual libraries before and after merging libraries.

**Classification of uniparental markers**

Mitochondrial reads were mapped to the revised Cambridge Reference Sequence (rCRS) mitochondrial genome (NC_012920.1). We obtained the mitochondrial mutations using GATK (v.3.7-0) HaplotypeCaller (25) and we further evaluated the haplotypes on IGV v.2.3 (26) to visually inspect any heteroplasmic positions. Haplogroup classification was made using HaploGrep 2.0 (27) following the nomenclature in PhyloTree (Build 17, February 2016) (28).

Haplotypes from male samples with Y-chromosome data were classified following with 2019 ISOGG list of mutations (International Society of Genetic Genealogy) using Yleaf (29). The Y-chromosome haplogroups were double-checked using pathPhynder default parameters (30).

**SNP calling from BAM files**

To call the variants we used two lists of SNPs: i) ~600k list used in the Human Origins dataset (Lazaridis et al., 2014) and ii) ~1240k SNPs included in the ‘1240k’ targeted enrichment protocol. The SNPs were called on all ancient samples using a combination of SAMtools mpileup and SequenceTools pileupCaller (with quality filters q20, Q20 enabled) (https://github.com/stschiff/sequenceTools). Pseudo-haploid genotypes were called by randomly choosing one allele from each site where there was read coverage, using pileupCaller.

**Relatedness determination**

To infer biological kinship relationships up to second degree we used the software Relationship Estimation from Ancient DNA (READ) (31). READ is optimised to manage low-coverage pseudo-diploid data. and is able to classify the type of relatedness degree as non-related, second degree (e.g. grandparent-grandchild, half-siblings, uncle/aunt, nephew/niece), first degree (parent-offspring, siblings), and identical (twins or duplicated individual). To confirm no cross-contamination happened between samples (which would show up as unexpected artificial relatedness), all combinations of samples were attempted regardless of location and time period. Only samples GOG34 and GOG35 were found to be biological relatives (1st degree).

**Data merging with public datasets**

In total, 53 libraries, corresponding to 12 individuals, passed endogenous DNA content and quality control thresholds.

We merged the newly generated ancient genomes reported here with genotypes from relevant (Figure S6) ancient and present-day populations from across the Mediterranean and neighbouring regions included in the Allen Ancient DNA resource dataset (32). We made use of modern populations from the dataset to compute the PCA onto where ancient individuals were projected (shrink mode and no outlier removal). We avoided comparisons with capture generated samples (which limited available sources from Iberia) to limit biases in *f*-statistics tests and *qpAdm*.

We also merged the dataset with a North African dataset from (33) for additional comparisons in *f*-statistics and expanding the diversity of North African groups. For haplotype-based analyses we relied on the 1000 Genomes Project publicly available data.

**Simulation of hybrid genomes**

We simulated hybrid genotypes of offspring between two parental sources with distinct ancestries, using present-day individuals from the Maghrebi and Iberian populations. The simulation followed Mendelian segregation rules for biallelic genotypes, and does not take into account recombination (Figure S4). If both parents are homozygous for the reference or alternative allele, then the hybrid offspring will carry two reference or alternate alleles respectively. If one parent is homozygous for allele A, and the other for allele B, then the offspring will always be assigned a heterozygous genotype. In situations where there could be more than one resulting genotype, a function assigned the genotype randomly according to the expected probabilities. We then projected these hybrids onto a PCA of a merged dataset with ~20k SNPs that included modern individuals from the HO dataset and Arauna *et al.* (33) dataset.

**Clustering analyses (PCA, ADMIXTURE)**

For principal component analysis we used the EIGENSOFT tool smartpca (34). The PCA dimensionality reduction was carried out using a subset of modern populations from the ~600k SNPs Human Origins dataset (35). The subset of modern populations included all individuals available from Europe, the Caucasus, Iran, the Near East, Arabia and North Africa. We projected 434 previously published ancient genomes from Spain, Anatolia, the Fertile Crescent and Morocco onto the PCA built with the modern populations (option lsqproject: YES).

We used the model-based clustering approach of ADMIXTURE (36) to estimate ancestry components in the newly generated samples, together with the same ancient individuals included in the PCA. We applied a filter for linkage disequilibrium in PLINK (--indep-pairwise 200, 25, 0.4), resulting in a total of ~200k LD-pruned positions used in the ADMIXTURE analysis. We ran ADMIXTURE in supervised (Figure 3A) and unsupervised mode (Figure S6). In unsupervised mode we only included ancient populations from *K*=2 to *K*=10 with the cross-validation flag activated (–cv). The lowest median CV error before a steep rise was for *K*=4. For the supervised ADMIXTURE, we chose Natufians, Iberomaurusians, Western Hunter-Gatherers and Caucasus Hunter-Gatherers as the source population because they can be identified as ultimate proxy sources of clearly differentiated ancestry found in all present-day Europeans. This is based on their age (e.g., distal admixed populations in time and space) and how they frame the corners of the PCA.

***F*-statistics**

Formal tests to evaluate treeness, gene flow and admixture were performed with *f*-statistics (37–39) included in the AdmixTools package v4.1 (40). Default parameters were used on the LD-pruned dataset. The *f*3 tests were used both in the form of outgroup-*f*3 and admixture-*f*3 depending on the scenario to be investigated.

We restricted the dataset to data generated using whole-genome shotgun sequencing to avoid potential biases with capture generated samples (which limited the available sources from Iberia) to avoid potential biases in *f*-statistics tests and *qpAdm*.

***qpAdm* modelling**

We used *qpWave/qpAdm* to test a variety of distal and more proximal models of ancestry (using <https://github.com/pontussk/qpAdm_wrapper>). We started by applying a modified version of a distal model optimised for post-Bronze Age West European populations first described in Patterson *et al.* (41), using *WHG.SG*, *Balkan_N.SG* and *Yamnaya.SG* as fixed sources to represent Western European Hunter-Gatherer-, Early European Farmer- and Steppe-related ancestries, respectively. Following the observation that the model is rejected for most individuals (Table S14) (*p*-values <0.05), we repeated the model adding Morocco_EN.SG to the source populations, as a proxy for North African-related ancestry.

To test more temporally proximal sources, we ran 1-source *qpWave* models, rotating through a reference list including outgroups and historical populations: *South_Africa_400BP.SG, Russia_Yana_UP.SG, Japan_Honshu_EarlyJomon.SG, Brazil_Sumidouro.SG, Morocco_EN.SG, Italy_RomanImperial.SG, Lebanon_Roman.SG, CanaryIslands_Guanche.SG, Portugal_Miroico_LateRoman.SG+Portugal_MonteDaNora_LateRoman.SG, England_IA_Roman.SG* (population labels as annotated in AADR v54).

For individuals with no single-source *qpWave* model accepted (all tested models with *p*-value <0.05) or with more than one model accepted (more than one model with *p*-value >0.05), we tested all possible combinations of two sources, using the same set of references.

**Analysis of imputed genomes**

For LAI, ROH (with the exception of hapROH) and heterozygosity analyses we made use of the imputed VCF files of the chromosomes. LAI was carried out with RFMix v2 (42). As reference haplotypes we used the phased VCFs from the 1000 Genomes Project together with the corresponding genetic maps.

**Runs of homozygosity**

We obtained information about ROH in different manners:

i) with hapROH (with default settings) for individuals GOG20, GOG23, GOG24, GOG26 and GOG50, and previously published individuals from southern Europe dating to the last 2500 years with a minimum of 400,000 SNPs overlapping with the ‘1240k’ SNP panel (2,6–8);

ii) with PLINK (with parameters --homozyg --homozyg-density 50 --homozyg-gap 100 --homozyg-kb 500 --homozyg-snp 50 --homozyg-window-het 1 --homozyg-window-snp 50 --homozyg-window-threshold 0.05) which only identifies certain segments of the chromosome as a ROH based on the stringency of the parameters given.

iii) by recording the length of each segment in between every single heterozygous position along the full extent of each chromosome. We call these segments, “organic ROH” (oROH) as opposed to the “PLINK ROH” since they are identified free of prior assumptions.

**Chromosome heterozygosity**

Sliding windows of heterozygosity along the chromosomes were computed after filtering and retaining transitions and transversions from the 1KPG Phase 3 list. We then divided his genome into 500 marker windows and calculated the percentage of heterozygous sites within each window, using an overlapping step of size 250 (43).

**Supplementary Tables list:**

- Table S1: Details of final sequencing results for the ancient samples studied.
- Table S2: Details of all samples screened for endogenous content.
- Table S3: Details of the new radiocarbon dates generated.
- Table S4: Homozygous segments identified in the imputed chromosomes using PLINK with default parameters.
- Table S5: Total number of RoH, total length of ROH measured in kb, and average length of ROH in kb. Includes the newly generated ancient genomes as well as the entire 1000 Genomes dataset.
- Table S6a: Measurements by sliding windows of number of variant sites in imputed chromosome 1 for the ancient genomes reported in this study. Used in Figure S8.
- Table S6b: Measurements by sliding windows of number of variant sites in imputed chromosome 11 for the ancient genomes reported in this study. Used in Figure S8.
- Table S6c: Measurements by sliding windows of number of variant sites in imputed chromosome 21 for the ancient genomes reported in this study. Used in Figure S8.
- Table S7a: Cumulative ROH curve for chromosome 1. Used in Figure S9.
- Table S7b: Cumulative ROH curve for chromosome 11. Used in Figure S9.
- Table S7c: Cumulative ROH curve for chromosome 21. Used in Figure S9.
- Table S8a: Organic ROH fragments in chromosome 1 identified in the ancient genomes reported in this study.
- Table S8b: Organic ROH fragments in chromosome 11 identified in the ancient genomes reported in this study.
- Table S8c: Organic ROH fragments in chromosome 21 identified in the ancient genomes reported in this study.
- Table S9: Summary statistics of organic ROH used for Figure 4B.
- Table S10: Shannon entropies for all imputed chromosomes in the ancient individuals reported.
- Table S11: Outgroup-f3 test with the medieval ancient genomes and modern populations merged overlapping the Allen Ancient DNA Resource (AADR 1240k) and Arauna et al., (2017) datasets. Used in Figure 2B.
- Table S12: Outgroup-f3 test for sample GOG50 with modern and ancient populations.
- Table S13: Proximal 1- and 2-source qpAdm models, rotating through a list of reference populations.
- Table S14: Distal qpAdm models with 3 and 4 fixed sources.
- Table S15: Genetic sex identification.

**Supplementary Figures**


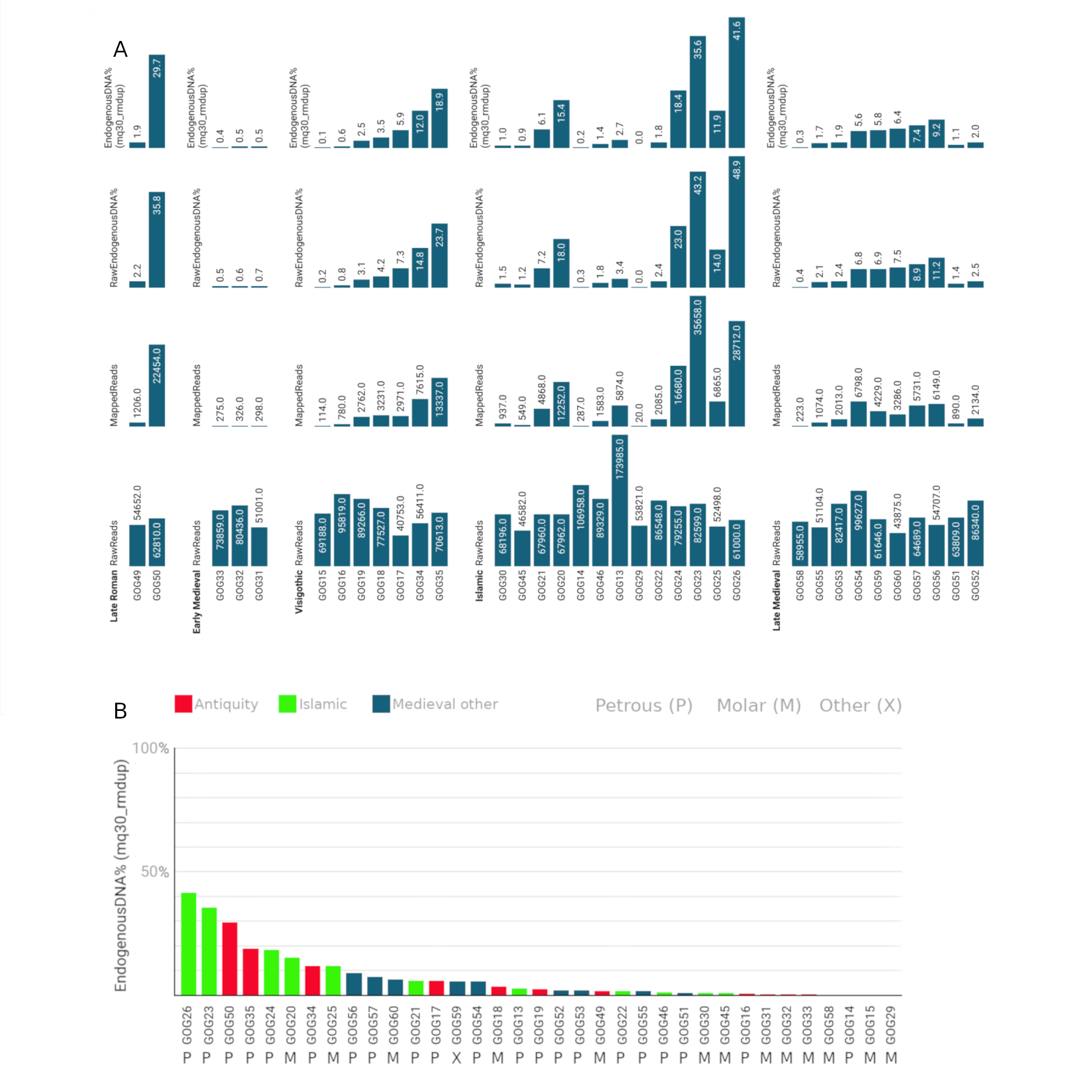


Fig. S1: A) Sequencing effort yield and details, this data was generated with a MiSeq platform during the screening process at Trinity College (Dublin) for all screened samples, ordered by sample and period. B) Adjusted endogenous DNA content after quality filters by sample, period and type of bone.


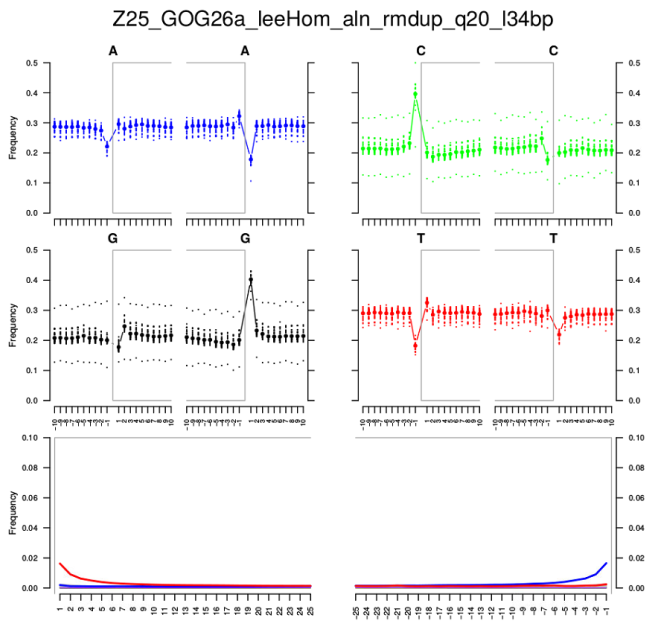


Fig. S2: Example of reduced damage pattern in UDG-treated library. All libraries for all samples presented the same reduced pattern of damage typical of ancient DNA. 3bp were soft-clipped in all reads based on the damage patterns seen in the treated libraries. Full damage patterns had been previously confirmed in non-treated screening libraries.


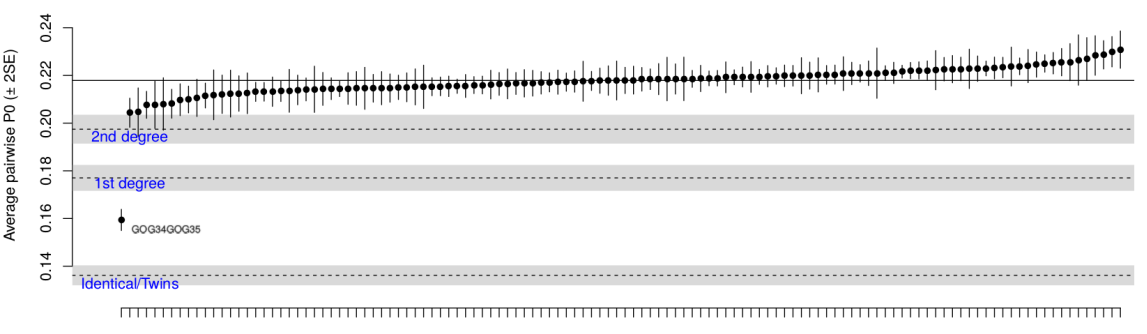


Fig. S3: READ pairwise kinship coefficients combinations for all newly reported ancient samples.


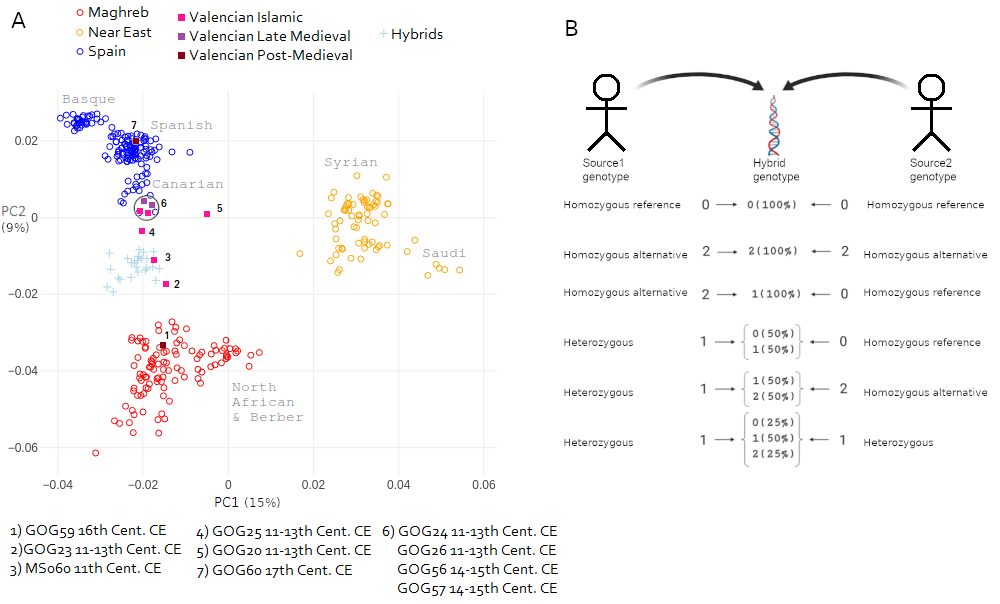


Fig. S4: PCA zoom-in with modern populations merged from the HO and Arauna *et al*. (33) datasets. Ancient medieval and simulated Spanish-Moroccan hybrid genomes projected.


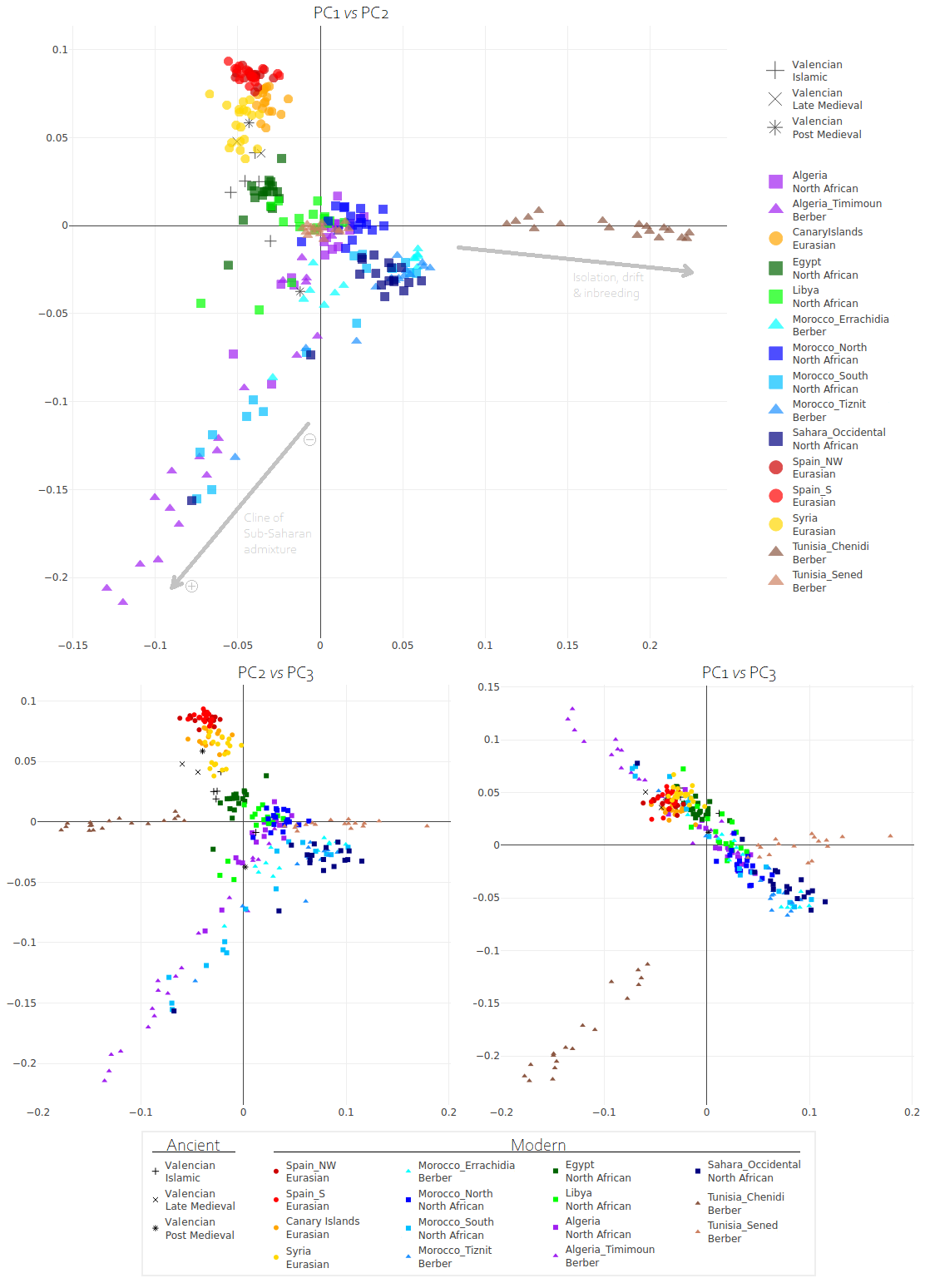


Fig. S5: PCA with *Arauna et al*. (33) dataset with the medieval samples projected.


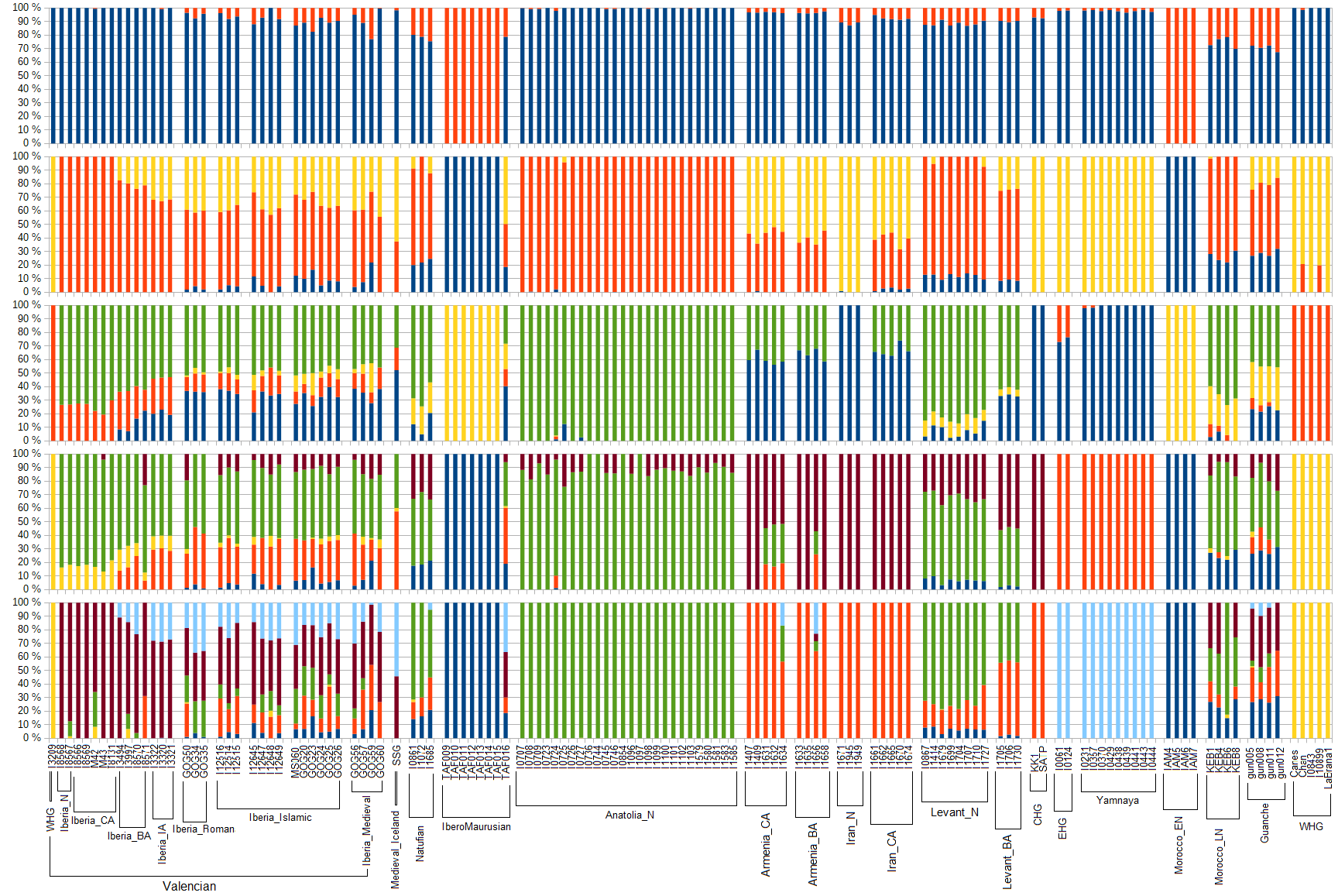


Fig. S6: Unsupervised ADMIXTURE using only ancient populations projected in Figure 2A (range of K values from K=2 to K=6).


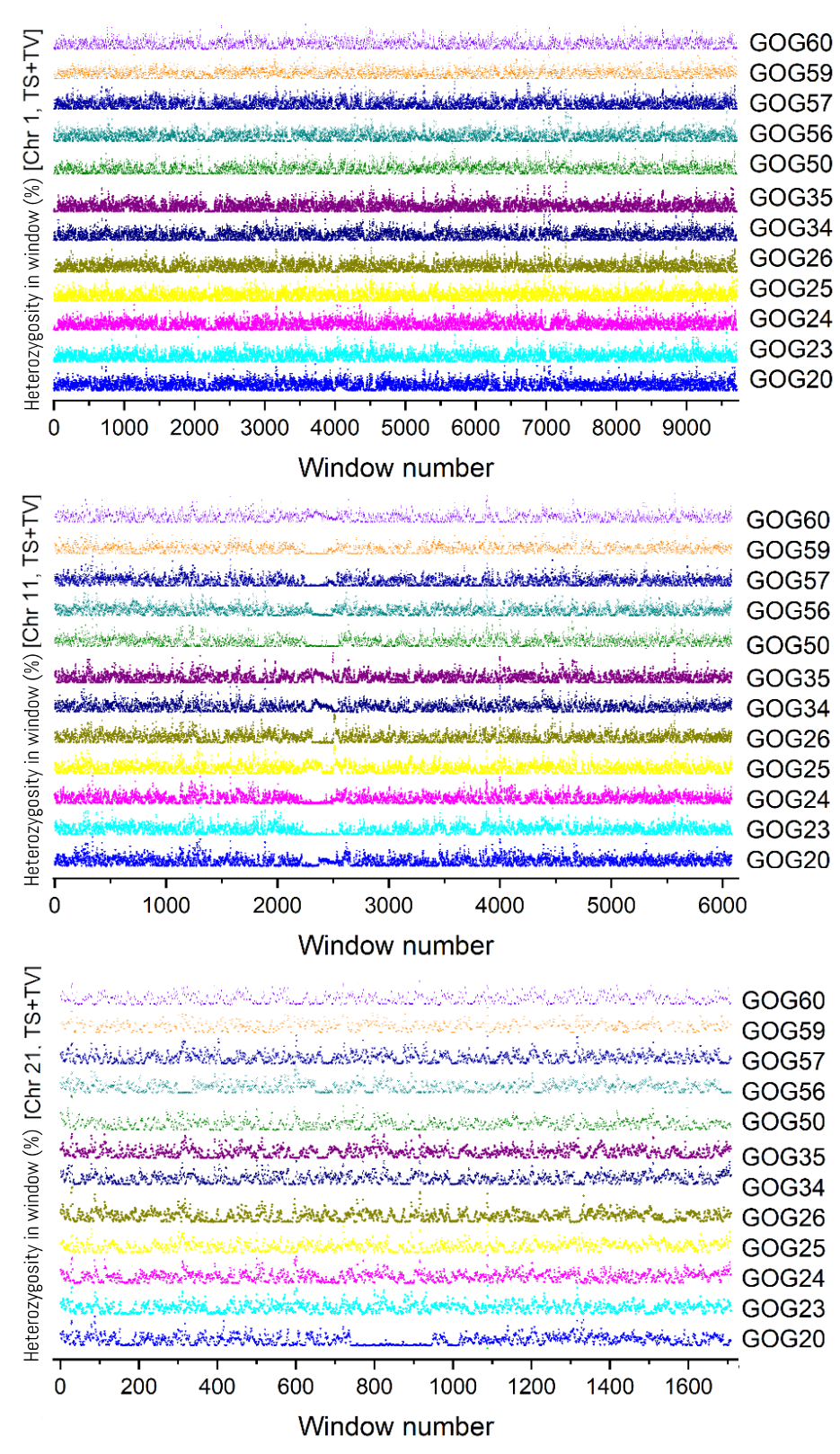


Fig. S7: Sliding window of heterozygosity along the length of all imputed chromosomes (chr1, chr11, chr21) in the ancient genomes reported in this study.


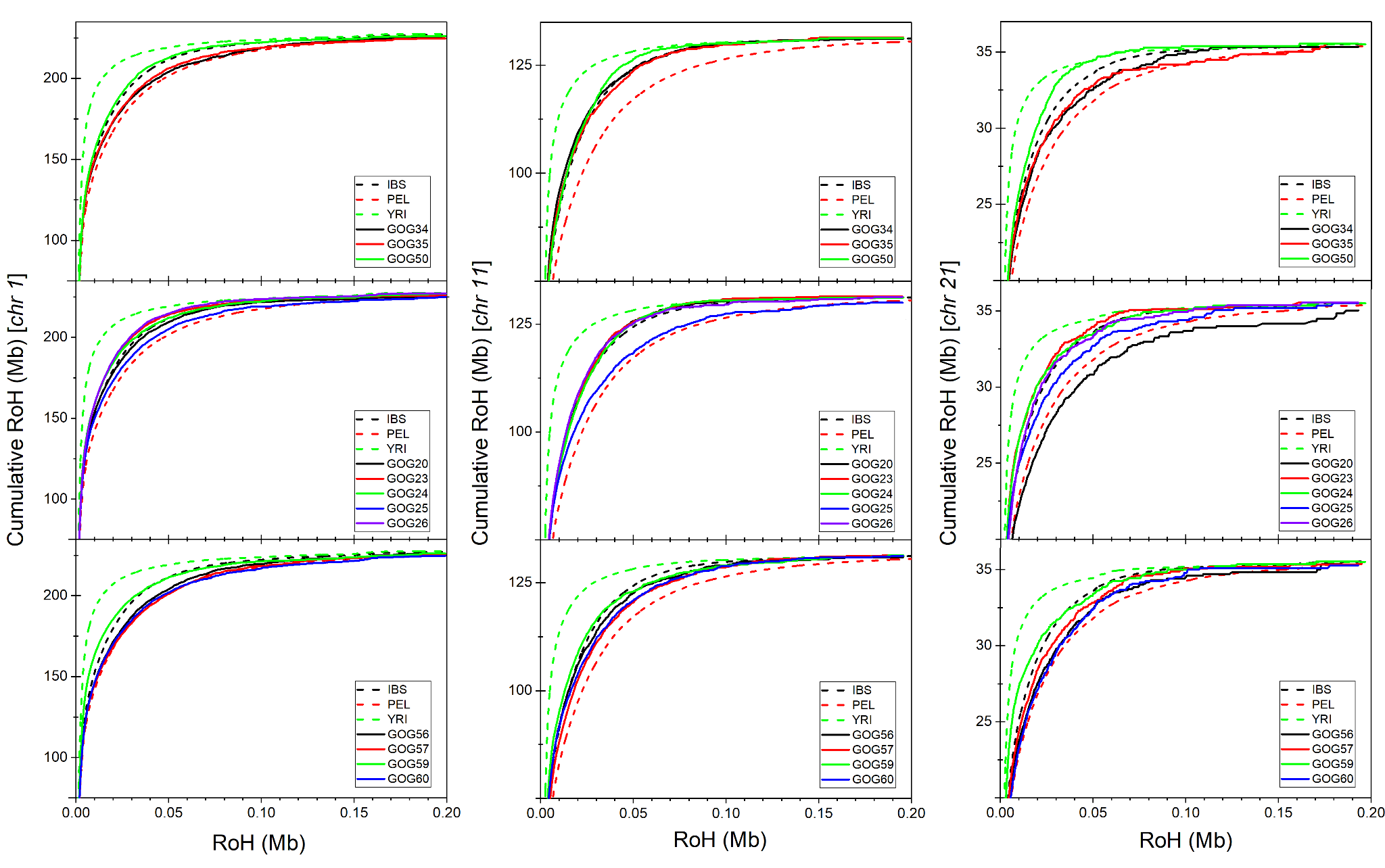


Fig. S8: Cumulative organic ROH for all imputed chromosomes (chr1, chr11, chr21) in the ancient genomes reported in this study.


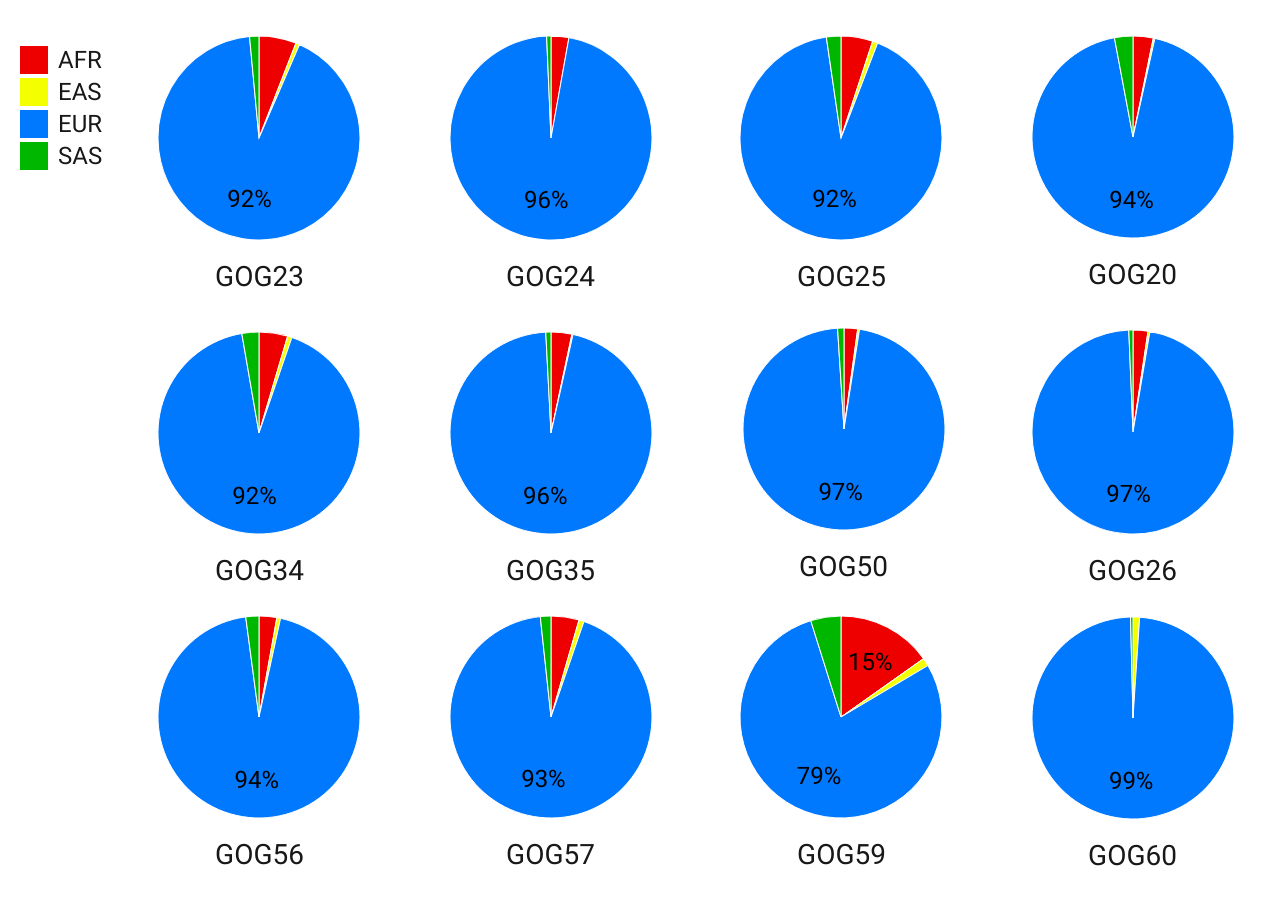


Fig. S9: Ancestry inferred using RFMix combining all imputed chromosomes (chr1, chr11, chr21) in the ancient genomes reported in this study.


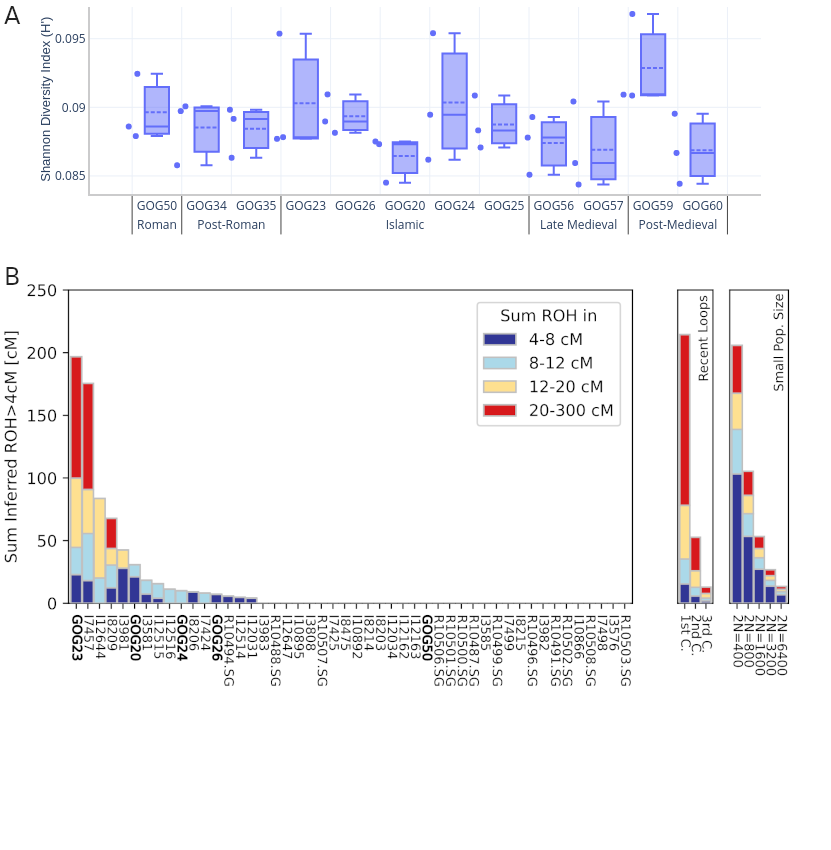


Fig. S10: A) Shannon diversity indexes calculated for each imputed chromosome (chr1, chr11, chr21) in the ancient genomes reported in this study using all of their variable positions. B) Extended comparison of hapROH results in figure 4C with other publicly available ancient individuals from Spain.


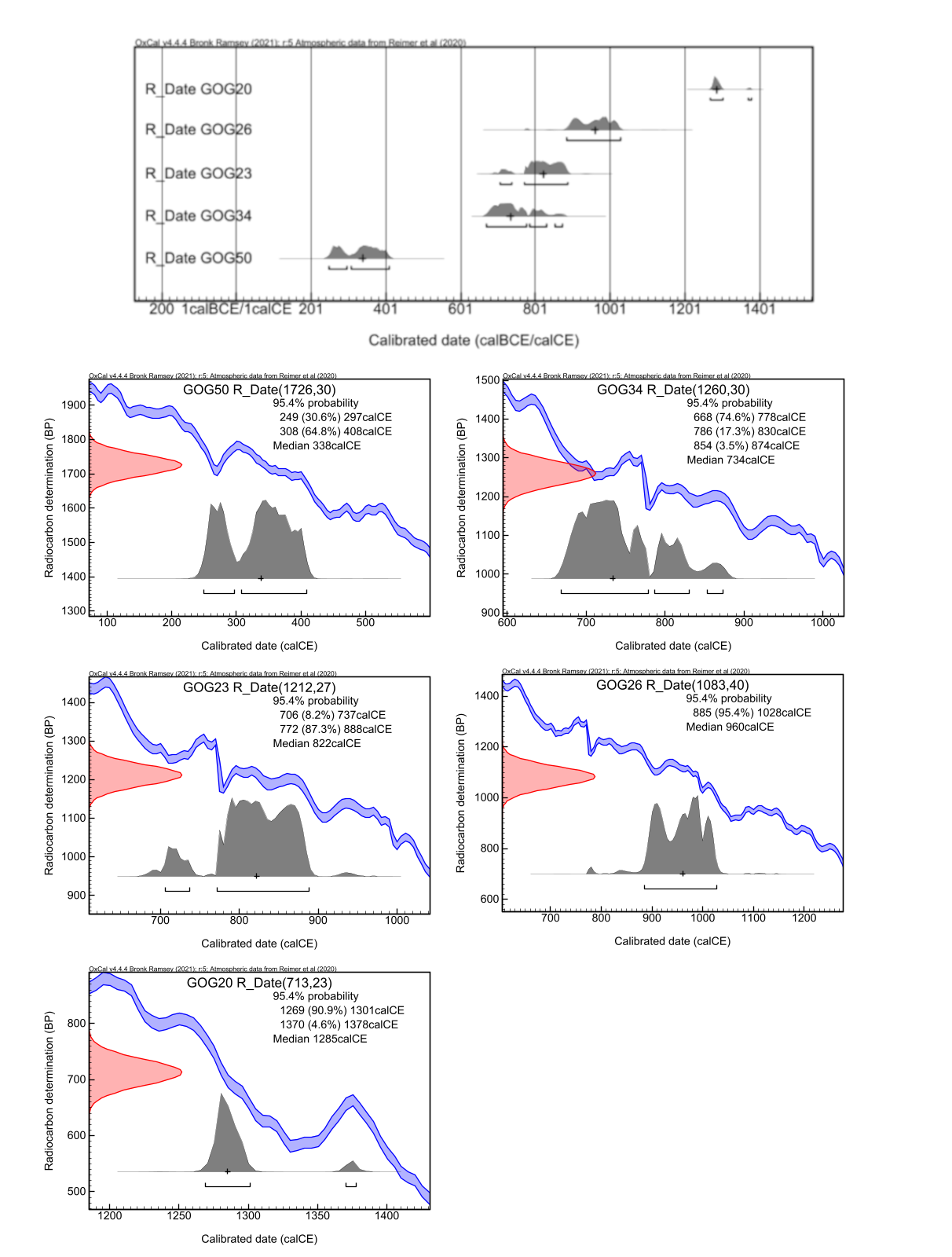


Fig. S11: Radiocarbon dates obtained for a subset of five samples reported in this study.


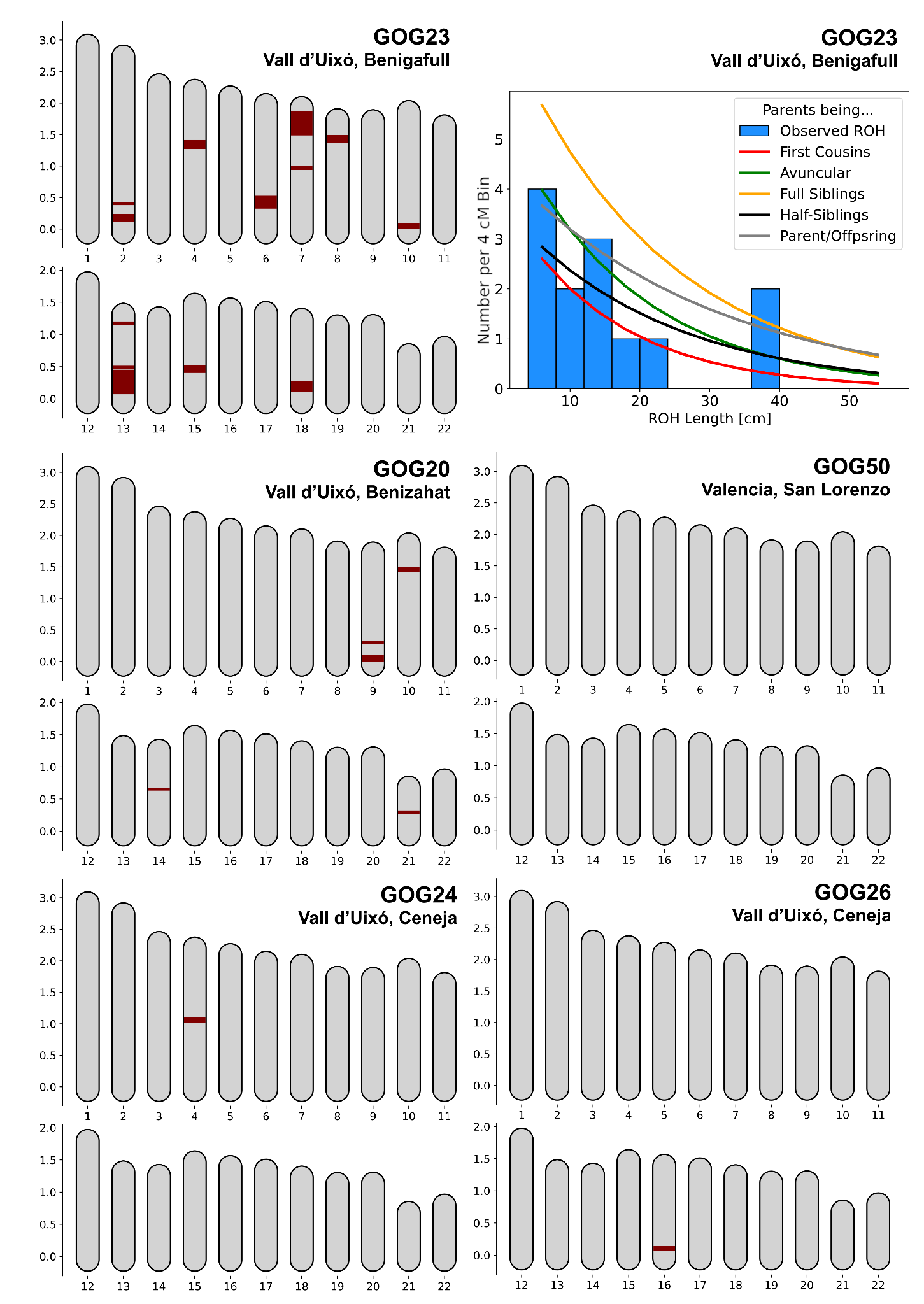


Fig. S12: karyotypes with ROH>4cM by chromosome (shown in red when present) identified with hapROH.

**Supplementary References**

1. Oteo-García G. Archaeogenetics of Southwest Europe [PhD Thesis]. University of Huddersfield; 2020. Available from: https://eprints.hud.ac.uk/id/eprint/35459

2. Olivé-Busom J, López-Costas O, Márquez-Grant N, Kirchner H. Estudio antropológico de las alquerías de Benizahat y Zeneta (Vall d’Uixó, Castellón). Una ventana a la vida rural andalusí. SAGVNTVM PLAV. 2021;53:193–212.

3. Oteo-García G, Alapont-Martín L, Pascual Beneyto J, Foody MGB, Yau B, Pala M, et al. Late Roman tombs at Sanxo Llop (Gandía, Valencia): Exogamy and kinship in a particular funerary structure. In: Death and the Societies of Late Antiquity: New methods, new questions? (Archéologies méditerranéennes). Aix-en-Provence: Presses universitaires de Provence; 2023. p. 119–27.

4. Silva M, Oteo-García G, Martiniano R, Guimarães J, von Tersch M, Madour A, et al. Biomolecular insights into North African-related ancestry, mobility and diet in eleventh-century Al-Andalus. Sci Rep. 2021;11(1):18121.

5. Pinhasi R, Fernandes D, Sirak K, Novak M, Connell S, Alpaslan-Roodenberg S, et al. Optimal ancient DNA yields from the inner ear part of the human petrous bone. PLoS ONE. 2015;10(6):e0129102.

6. Bronk Ramsey C. Bayesian analysis of radiocarbon dates. Radiocarbon. 2009;51(1):337–60.

7. Reimer P, Austin W, Bard E, Bayliss A, Blackwell P, Bronk Ramsey C, et al. The IntCal20 Northern hemisphere radiocarbon age calibration Curve (0–55 cal kBP). Radiocarbon. 2020;62(4):725–57.

8. Rohland N, Hofreiter M. Ancient DNA extraction from bones and teeth. Nat Protoc. 2007;2(7):1756–62.

9. Yang DY, Eng B, Waye JS, Dudar JC, Saunders SR. Improved DNA extraction from ancient bones using silica-based spin columns. Am J Phys Anthropol. 1998;105(4):539–43.

10. MacHugh D, Edwards C, Bailey J, Bancroft D, Bradley D. The extraction and analysis of ancient DNA from bone and teeth: a survey of current methodologies. Anc Biomol. 2000;3.

11. Meyer M, Kircher M, Gansauge MT, Li H, Racimo F, Mallick S, et al. A high-coverage genome sequence from an archaic Denisovan individual. Science. 2012;338(6104):222–6.

12. Gamba C, Hanghøj K, Gaunitz C, Alfarhan AH, Alquraishi SA, Al-Rasheid KAS, et al. Comparing the performance of three ancient DNA extraction methods for high-throughput sequencing. Mol Ecol Resour. 2016;16(2):459–69.

13. Cassidy LM, Martiniano R, Murphy EM, Teasdale MD, Mallory J, Hartwell B, et al. Neolithic and Bronze Age migration to Ireland and establishment of the insular Atlantic genome. Proc Natl Acad Sci. 2016;113(2):368–73.

14. Briggs AW, Stenzel U, Johnson PLF, Green RE, Kelso J, Prüfer K, et al. Patterns of damage in genomic DNA sequences from a Neandertal. Proc Natl Acad Sci U S A. 2007;104(37):14616–21.

15. Briggs AW, Stenzel U, Meyer M, Krause J, Kircher M, Pääbo S. Removal of deaminated cytosines and detection of in vivo methylation in ancient DNA. Nucleic Acids Res. 2009;38(6):e87.

16. Lindahl T. Instability and decay of the primary structure of DNA. Nature. 1993;362(6422):709–15.

17. Lindahl T. The Croonian Lecture, 1996: Endogenous damage to DNA. Philos Trans R Soc B Biol Sci. 1996;351(1347):1529–38.

18. Renaud G, Stenzel U, Kelso J. LeeHom: Adaptor trimming and merging for Illumina sequencing reads. Nucleic Acids Res. 2014;

19. García-Alcalde F, Okonechnikov K, Carbonell J, Cruz LM, Götz S, Tarazona S, et al. Qualimap: evaluating next-generation sequencing alignment data. Bioinformatics. 2012;28(20):2678–9.

20. Martiniano R, Cassidy LM, Ó’Maoldúin R, McLaughlin R, Silva NM, Manco L, et al. The population genomics of archaeological transition in west Iberia: Investigation of ancient substructure using imputation and haplotype-based methods. PLoS Genet. 2017;13(7).

21. Rohland N, Harney E, Mallick S, Nordenfelt S, Reich D. Partial uracil – DNA – glycosylase treatment for screening of ancient DNA. Philos Trans R Soc B Biol Sci. 2015;370(1660):20130624.

22. Jónsson H, Ginolhac A, Schubert M, Johnson PLF, Orlando L. MapDamage2.0: Fast approximate Bayesian estimates of ancient DNA damage parameters. In: Bioinformatics. 2013. p. 1682–4.

23. Malaspinas AS, Tange O, Moreno-Mayar JV, Rasmussen M, DeGiorgio M, Wang Y, et al. bammds: a tool for assessing the ancestry of low-depth whole-genome data using multidimensional scaling (MDS). Bioinforma Oxf Engl. 2014;30(20):2962–4.

24. Skoglund P, Storå J, Götherström A, Jakobsson M. Accurate sex identification of ancient human remains using DNA shotgun sequencing. J Archaeol Sci. 2013;40(12):1427–32.

25. McKenna A, Hanna M, Banks E, Sivachenko A, Cibulskis K, Kernytsky A, et al. The genome analysis toolkit: A MapReduce framework for analyzing next-generation DNA sequencing data. Genome Res. 2010;20(9):1297–303.

26. Thorvaldsdóttir H, Robinson JT, Mesirov JP. Integrative Genomics Viewer (IGV): High-performance genomics data visualization and exploration. Brief Bioinform. 2013;14(2):178–92.

27. Weissensteiner H, Pacher D, Kloss-Brandstätter A, Forer L, Specht G, Bandelt HJ, et al. HaploGrep 2: mitochondrial haplogroup classification in the era of high-throughput sequencing. Nucleic Acids Res. 2016;44(W1):W58–63.

28. van Oven M. PhyloTree Build 17: Growing the human mitochondrial DNA tree. Forensic Sci Int Genet Suppl Ser. 2015;5:E392–4.

29. Ralf A, Montiel González D, Zhong K, Kayser M. Yleaf: Software for human Y-Chromosomal haplogroup Inference from next-generation sequencing data. Mol Biol Evol. 2018;35(5):1291–4.

30. Martiniano R, De Sanctis B, Hallast P, Durbin R. Placing ancient DNA sequences into reference phylogenies. Mol Biol Evol. 2022;39(2):msac017.

31. Monroy Kuhn JM, Jakobsson M, Günther T. Estimating genetic kin relationships in prehistoric populations. PLoS ONE. 2018;13(4):e0195491.

32. Mallick S, Reich D. The Allen Ancient DNA Resource (AADR): A curated compendium of ancient human genomes. 2023.

33. Arauna LR, Mendoza-Revilla J, Mas-Sandoval A, Izaabel H, Bekada A, Benhamamouch S, et al. Recent historical migrations have shaped the gene pool of Arabs and berbers in North Africa. Mol Biol Evol. 2017;34(2):318–29.

34. Patterson N, Price AL, Reich D. Population structure and eigenanalysis. PLoS Genet. 2006 Dec;2(12):2074–93.

35. Lazaridis I, Patterson N, Mittnik A, Renaud G, Mallick S, Kirsanow K, et al. Ancient human genomes suggest three ancestral populations for present-day Europeans. Nature. 2014;513:409–13.

36. Alexander DH, Novembre J, Lange K. Fast model-based estimation of ancestry in unrelated individuals. Genome Res. 2009;19(9):1655–64.

37. Green RE, Krause J, Briggs AW, Maricic T, Stenzel U, Kircher M, et al. A draft sequence of the neandertal genome. Science. 2010;328(5979):710–22.

38. Reich D, Thangaraj K, Patterson N, Price AL, Singh L. Reconstructing Indian population history. Nature. 2009;461(7263):489–94.

39. Peter BM. Admixture, population structure, and f-statistics. Genetics. 2016;202(4):1485–501.

40. Patterson N, Moorjani P, Luo Y, Mallick S, Rohland N, Zhan Y, et al. Ancient admixture in human history. Genetics. 2012;192:1065–93.

41. Patterson N, Isakov M, Booth T, others. Large-scale migration into Britain during the Middle to Late Bronze Age. Nature. 2022;601:588–94.

42. Maples BK, Gravel S, Kenny EE, Bustamante CD. RFMix: a discriminative modeling approach for rapid and robust local-ancestry inference. Am J Hum Genet. 2013;93(2):278–88.

43. Cassidy LM, Maoldúin RÓ, Kador T, Lynch A, Jones C, Woodman PC, et al. A dynastic elite in monumental Neolithic society. Nature. 2020;582:384–8.
