# Supplementary material for: Medieval genomes from eastern Iberia illuminate the role of Morisco mass deportations in dismantling a long-standing genetic bridge with North Africa
Source: Genome Biol. 2025 Apr 28;26:108. doi: 10.1186/s13059-025-03570-1 (PMC12036142; doi:10.1186/s13059-025-03570-1)
Supplement: Supplementary file 3 — Additional file 3: Raw data at ENA information. [file 13059_2025_3570_MOESM3_ESM.docx]

**RAW DATA INFORMATION**

**ENA_ID ACCESSION LIBRARY_ID LAB_ID**

ERS23851376 PRJEB65253 Z13_GOG20a GOG20

ERS23851378 PRJEB65253 Z14_GOG20b GOG20

ERS23851380 PRJEB65253 Z15_GOG20c GOG20

ERS23851382 PRJEB65253 Z16_GOG23a GOG23

ERS23851384 PRJEB65253 Z17_GOG23b GOG23

ERS23851386 PRJEB65253 Z18_GOG23c GOG23

ERS23851388 PRJEB65253 Z19_GOG24a GOG24

ERS23851390 PRJEB65253 Z20_GOG24b GOG24

ERS23851392 PRJEB65253 Z21_GOG24c GOG24

ERS23851394 PRJEB65253 Z22_GOG25a GOG25

ERS23851396 PRJEB65253 Z23_GOG25b GOG25

ERS23851398 PRJEB65253 Z24_GOG25c GOG25

ERS23851400 PRJEB65253 Z25_GOG26a GOG26

ERS23851402 PRJEB65253 Z26_GOG26b GOG26

ERS23851404 PRJEB65253 Z27_GOG26c GOG26

ERS23851406 PRJEB65253 Z28_GOG34a GOG34

ERS23851408 PRJEB65253 Z29_GOG34b GOG34

ERS23851410 PRJEB65253 Z30_GOG34c GOG34

ERS23851412 PRJEB65253 Z31_GOG35a GOG35

ERS23851414 PRJEB65253 Z32_GOG35b GOG35

ERS23851416 PRJEB65253 Z33_GOG35c GOG35

ERS23851418 PRJEB65253 Z37_GOG501a GOG50

ERS23851420 PRJEB65253 Z38_GOG501b GOG50

ERS23851422 PRJEB65253 Z39_GOG501c GOG50

ERS23851424 PRJEB65253 Z40_GOG502a GOG50

ERS23851426 PRJEB65253 Z41_GOG502b GOG50

ERS23851428 PRJEB65253 Z42_GOG502c GOG50

ERS23851430 PRJEB65253 Z43_GOG56a GOG56

ERS23851432 PRJEB65253 Z44_GOG56b GOG56

ERS23851434 PRJEB65253 Z45_GOG56c GOG56

ERS23851436 PRJEB65253 Z46_GOG57a GOG57

ERS23851438 PRJEB65253 Z47_GOG57b GOG57

ERS23851440 PRJEB65253 Z48_GOG57c GOG57

ERS23851442 PRJEB65253 Z49_GOG59a GOG59

ERS23851444 PRJEB65253 Z50_GOG59b GOG59

ERS23851446 PRJEB65253 Z51_GOG59c GOG59

ERS23851448 PRJEB65253 Z52_GOG60a GOG60

ERS23851450 PRJEB65253 Z53_GOG60b GOG60
